# Transcriptome Sequencing and Expression Analysis of Terpenoid Biosynthesis Genes in *Litsea cubeba*

**DOI:** 10.1371/journal.pone.0076890

**Published:** 2013-10-09

**Authors:** Xiao-Jiao Han, Yang-Dong Wang, Yi-Cun Chen, Li-Yuan Lin, Qing-Ke Wu

**Affiliations:** 1 State Key Laboratory of Forest Genetics and Tree Breeding, Chinese Academy of Forestry, Beijing, P. R. China; 2 Research Institute of Subtropical Forestry, Chinese Academy of Forestry, Fuyang, P. R. China; Kyushu Institute of Technology, Japan

## Abstract

**Background:**

Aromatic essential oils extracted from fresh fruits of *Litsea cubeba* (Lour.) Pers., have diverse medical and economic values. The dominant components in these essential oils are monoterpenes and sesquiterpenes. Understanding the molecular mechanisms of terpenoid biosynthesis is essential for improving the yield and quality of terpenes. However, the 40 available *L. cubeba* nucleotide sequences in the public databases are insufficient for studying the molecular mechanisms. Thus, high-throughput transcriptome sequencing of *L. cubeba* is necessary to generate large quantities of transcript sequences for the purpose of gene discovery, especially terpenoid biosynthesis related genes.

**Results:**

Using Illumina paired-end sequencing, approximately 23.5 million high-quality reads were generated. *De* *novo* assembly yielded 68,648 unigenes with an average length of 834 bp. A total of 38,439 (56%) unigenes were annotated for their functions, and 35,732 and 25,806 unigenes could be aligned to the GO and COG database, respectively. By searching against the Kyoto Encyclopedia of Genes and Genomes Pathway database (KEGG), 16,130 unigenes were assigned to 297 KEGG pathways, and 61 unigenes, which contained the mevalonate and 2-C-methyl-D-erythritol 4-phosphate pathways, could be related to terpenoid backbone biosynthesis. Of the 12,963 unigenes, 285 were annotated to the terpenoid pathways using the PlantCyc database. Additionally, 14 terpene synthase genes were identified from the transcriptome. The expression patterns of the 16 genes related to terpenoid biosynthesis were analyzed by RT-qPCR to explore their putative functions.

**Conclusion:**

RNA sequencing was effective in identifying a large quantity of sequence information. To our knowledge, this study is the first exploration of the *L. cubeba* transcriptome, and the substantial amount of transcripts obtained will accelerate the understanding of the molecular mechanisms of essential oils biosynthesis. The results may help improve future genetic and genomics studies on the molecular mechanisms behind the chemical composition of essential oils in *L. cubeba* fruits.

## Introduction

*Litsea cubeba* (Lour.) Pers (mountain pepper). an evergreen or deciduous dioecious tree or shrub, belongs to a species of the genus Litsea in the Lauraceae family. It is a tropical and subtropical plant distributed in southeastern Asia, southern China, Japan, and Taiwan. The fruit and bark of *L. cubeba* are commonly used as traditional herbal medicines for the treatment of stomachaches, cold, hiccups, inflammation, gastric cavity crymodynia, headaches, coronary heart disease and atopic eczema [1,2]. Aromatic essential oils extracted from the fresh fruits are flavor enhancers in foods, cosmetics, and cigarettes [3]. At the same time, the essential oils of *L. cubeba* exhibit a range of bioactivities such as antioxidant [4], antitermite [5], larvicidal [6], cytotoxic [7], neuropharmacological [8] and antimicrobial [9] activities. *L. cubeba* is a genetically diploid (2n=2x=24) plant [10]. Despite the medicinal and economic importance of *L. cubeba*, little genomic information is available for this non-model genus, and only 40 nucleotide sequences have been deposited in the NCBI GenBank database (as of the 22^nd^ of May 2013). To date, three genes involved in monoterpene synthases have been functionally characterized [11], showing the potential for genomic research on this plant. However, a lack of sequence data has limited extensive and intensive research. Therefore, it is necessary to explore genomic data sources of *L. cubeba* for gene discovery and further functional studies.

In the essential oils of *L. cubeba*, the dominant components are monoterpenes represented mainly by neral and geranial, which are *cis-*trans isomers of citral [12]. In addition, it also contains sesquiterpene and other non-terpenes [7,12]. The universal precursors for terpenoids are two branched unsaturated diphosphate isoprene units, isopentenyl pyrophosphate (IPP) and dimethylallyl pyrophosphate (DMAPP), which come from the mevalonate (MVA) and 2-C-methyl-Derythritol 4-phosphate (MEP) pathways, respectively [13,14]. IPP and DMAPP can be converted into each other by IPP isomerase (IPPI) [15]. IPP and DMAPP are catalyzed to form geranyl diphosphate (GPP) by GPP synthase, while IPP is converted into farnesyl diphosphate (FPP) by FPP synthase [16]. Subsequently, monoterpenes and sesquiterpenes are generated from the precursors GPP and FPP through the action of the monoterpene synthase (mono-TPS) and sesquiterpene synthase (sesqui-TPS), respectively [17]. However, it is difficult to differentiate the induction patterns of the individual TPSs due to the high level of sequence identity among TPS family members, which indicates the rapid evolution of a species-specific paralogous gene cluster [18]. The discovery and identification of key genes responsible for the terpenoid biosynthesis in *L. cubeba* could help regulate the composition of essential oils.

RNA sequencing (RNA-seq) has become a powerful technology to profile transcriptomes due to its high-throughput, accuracy, and reproducibility [19]. In plants, RNA-seq has accelerated the investigation of the complexity of gene transcription patterns, functional analyses and gene regulation networks [20]. Due to the limited genomic sources for *L. cubeba*, it is important to identify whole transcripts for complete gene expression profiling by RNA-seq. In the present work, an RNA-seq project for *L. cubeba* was initiated. Eleven RNA samples, including various tissues and fruits of different development and ripening stages, were sequenced using the high-throughput Illumina deep sequencing technique. In addition, we estimated the expression profiles of key genes responsible for terpenoid biosynthesis. The transcriptome sequencing from *L. cubeba* may help improve future genetic and genomics studies on the molecular mechanisms behind the chemical composition of the fruit’s essential oils.

## Results

### RNA-Seq and *de novo* transcriptome assembly

In previous works, the *de novo* assembly of short reads without a reference genome was a challenge despite the development of many bioinformatics software tools for data assembly and analysis [21,22]. To maximize the range of transcript diversity, a mixed RNA sample from four tissues and seven different developmental stages of fruits was prepared for RNA-seq using the Illumina HiSeq^TM^ 2000. After a stringent quality check, we obtained by sequencing 3.66 million raw reads and 6.66 gigabase pairs (Gbp) with an average GC content of 47.66% (File S1). We defined the reads with *Q*≥20 and no ambiguous “N” as high-quality reads. After the removal of adaptor sequences and exclusion of contaminated or short reads, 23,460,490 high-quality reads were assembled into 1,973,896 contigs (Table 1) using SOAPdenovo [23]. Using the Trinity *de novo* assembly program, next-generation short-read sequences were assembled into 99,060 transcripts with a mean length of 680.34 bp. The transcripts were subjected to cluster and assembly analyses. Finally, we harvested a total of 68,648 unigenes with an average length of 834 bp, which included 10,270 unigenes (14.96%) with lengths greater than 1 kb. These results showed that the throughput and sequencing quality was high enough for the following analyses.

**Table 1:** Length distribution of assembled contigs, transcripts, and unigenes.

Nucleotide length (bp)	Contigs	Transcripts	Unigenes
0-100	1,758,303	0	0
100-200	134,073	0	0
200-300	34,458	33,002	28,329
300-400	14,452	15,481	12,137
400-500	7,512	8,861	6,134
500-600	4,722	6,157	3,822
600-700	3,256	4,621	2,678
700-800	2,578	3,923	2,100
800-900	2,047	3,319	1,732
900-1000	1,697	2,921	1,446
1000-1100	1,475	2,648	1,284
1100-1200	1,296	2,305	1,158
1200-1300	1,111	2,057	1,019
1300-1400	844	1,773	839
1400-1500	824	1,579	799
1500-1600	731	1,364	663
1600-1700	650	1,199	615
1700-1800	577	1,057	553
1800-1900	465	978	455
1900-2000	407	804	424
2000-2100	390	719	379
2100-2200	322	638	316
2200-2300	267	524	261
2300-2400	194	424	202
2400-2500	202	420	193
2500-2600	176	363	183
2600-2700	121	262	126
2700-2800	91	230	115
2800-2900	87	191	86
2900-3000	72	174	82
3000-3100	71	146	71
>3000	425	920	447
Total number	1,973,896	99,060	68,648
Total length	132,955,711	67,394,111	39,398,812
N50 length	87	1053	834
Mean length	67.35699905	680.3362709	573.9251253

**Figure 1:**
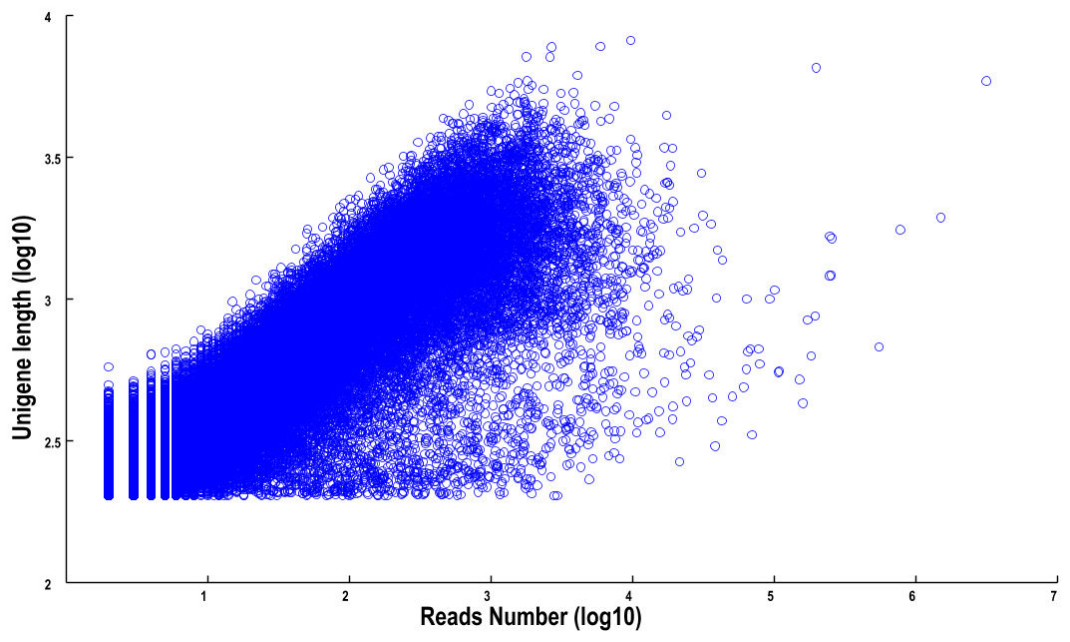
The dependence of unigene lengths on the number of reads assembled into each unigene.

The length distributions of contigs, transcripts and unigenes are shown in Table 1, revealing that the distribution of transcripts showed a similar tendency to that of the unigenes. The N50 values of transcripts and unigenes were 1053 bp and 834 bp, respectively. As expected for a randomly fragmented transcriptome, there was a positive relationship between the length of a given unigene and the number of reads (Figure 1). In addition, we made an Open Reading Frame (ORF) prediction analysis. ORFs were predicted using getORF from the EMBOSS package and most unigenes (99.5%) were identified as having ORFs starting at an ‘ATG’ codon. To facilitate the access and use of the *L. cubeba* transcriptome sequencing data, the raw paired-end sequence data in the FASTQ format was deposited in the National Center for Biotechnology Information (NCBI) Sequence Read Archive (SRA) database with accession number SRA080286.

**Figure 2:**
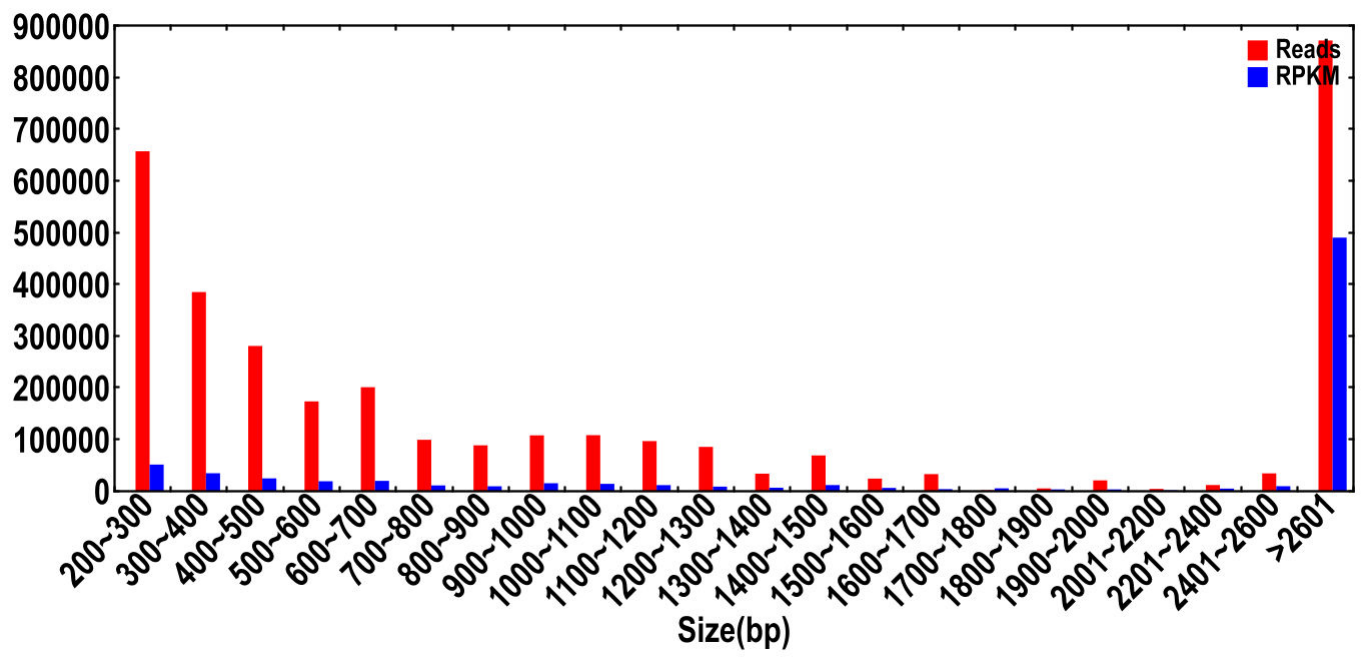
Assessment of assembly quality. Distribution of unique-mapped reads and RPKM (reads per kb per million reads) of the assembled unigenes.

Using the number of reads per kilobase of exon model per million mapped reads (RPKM) approach, the expression of each unigene was estimated by reading the depth of deep sequencing. According to Figure 2, the expression of each unigene varied similarly with sequencing depth. The expression of unigenes ranged from 0 to 22,608 RPKM with an average of 18 RPKM. Of 65,535 unigenes, 54,734 (83.5%) had a very low expression level of less than 10 RPKM. In addition, we found that most of the unigenes with high RPKM values were seed storage proteins, such as albumin, globulin and vicilin-like embryo storage protein. A higher proportion of fruits (seven different ripening stages) in the 11 samples may be an explanation for this result.

### Functional annotation and classification

**Figure 3:**
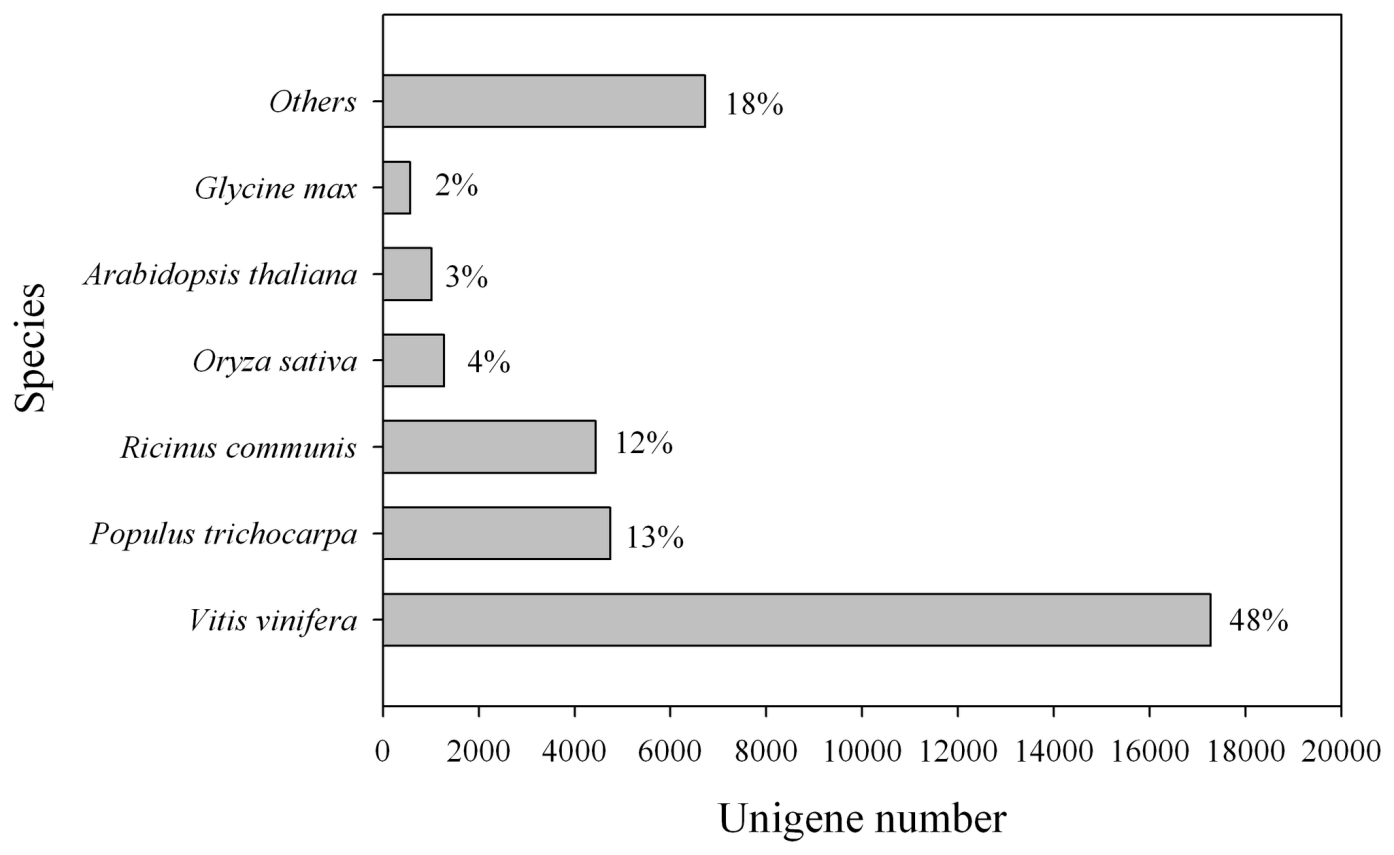
Species distribution of the top BLAST hits in Nr dababase. 36,041 BLASTX-hit unigenes were calculated. Species with proportions of more than 1% are shown.

Only 56% of unigenes (38,439) were annotated with a threshold of 10^-5^ by performing a BLASTX search against diverse protein databases, including the NCBI nonredundant protein (Nr) database, NCBI non-redundant nucleotide sequence (Nt) database, UniProt/Swiss-Prot, Kyoto Encyclopedia of Genes and Genomes (KEGG), Cluster of Orthologous Groups of proteins (COG), UniProt/TrEMBL and PlantCyc database. The overall functional annotation for *L. cubeba* is listed in Table 2. According to the BLASTX results, 36,041 (52.5%) unigenes have homologous proteins in the Nr protein database. Interestingly, 17,272 (48%) unigenes showed significant homology with sequences of *Vitis vinifera*, and 13% and 12% of the mapped sequences have a high similarity with sequences of *Populus trichocarpa* and *Ricinus communis*, respectively (Figure 3). Furthermore, 25,806 (37.6%) unigenes had significant matches in the Nt database, and 25,606 (37.3%) unigenes had similarity to proteins in the Swiss-Prot database.

**Table 2:** Functional annotation of the *L. cubeba.*

Annotated databases	Unigenes	Percentage of unigenes
Nr-annotation	36,041	52.5%
Nt-annotation	25,806	37.6%
SwissProt-annotation	25,606	37.3%
TrEMBL-annotation	35,732	52.1%
GO-annotation	25,340	36.9%
KEGG-annotation	7,702	11.2%
COG-annotation	9,803	14.3%
PlantCyc-annotation	12,963	18.9%
Total	38,439	56.0%

**Figure 4:**
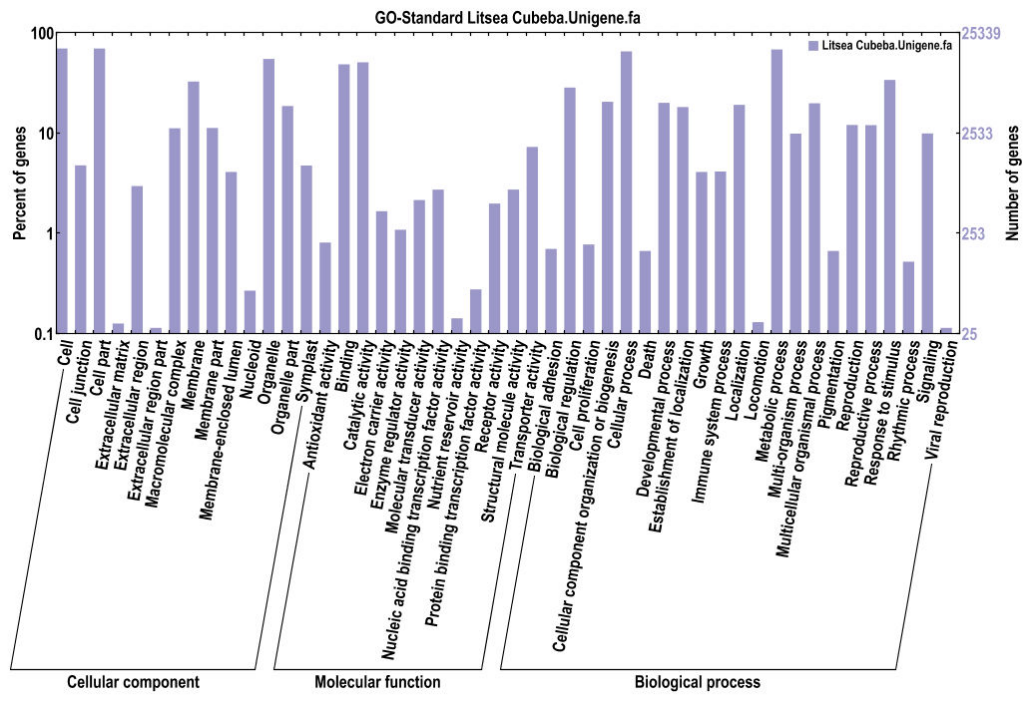
Functional annotation of assembled sequences based on gene ontology (GO) categorization. The unigenes are summarized into three main categories: cellular component, molecular function and biological process.

Generally, GO was used to classify the functions of the assembled transcripts and describe gene products in terms of their associated biological processes, cellular components, and molecular functions. A total of 25,340 unigenes were distributed into the three categories with 46.28% in biological processes, 15.93% in molecular functions, and 37.79% in cellular components (Figure 4). To better review GO classification, each GO term was further clustered to its parent term. The results showed that the three largest biological processes were cellular processes, metabolic processes, and response to stimulus (Figure 4). Meanwhile, most of the genes were classified into the molecular functions of binding, catalytic activity, and transporter activity. In cellular components, the major classifications for these gene products were cell, cell part, and organelle. The results indicated that most of the sequenced genes were responsible for fundamental biological regulation and metabolism.

**Figure 5:**
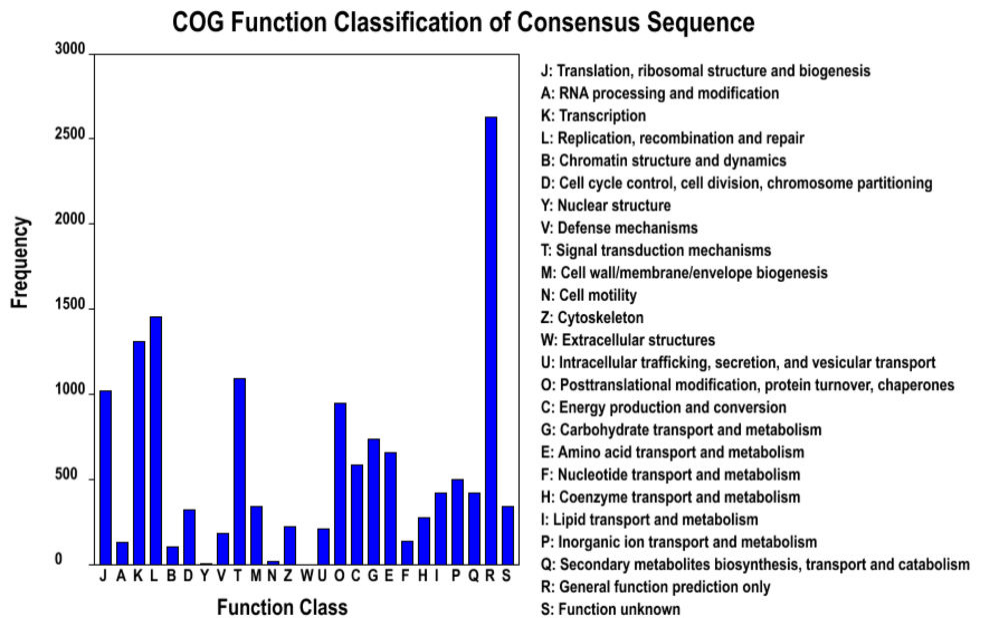
Clusters of orthologous groups (COG) classification. In total, 25,806 of the 68,648 sequences with Nr hits were grouped into 25 classifications.

Based on the Nr annotation, only 9,803 of 68,648 (14.3%) unigenes could be aligned to the COG database to predict and classify possible functions. According to the annotation of COG, these genes were classified into 25 different functional classes, including RNA processing and modification, energy production and conversion, carbohydrate transport and metabolism, signal transduction mechanisms, lipid transport and metabolism, coenzyme transport and metabolism (Figure 5). The cluster for general function prediction (2,629; 18.76%) represented the largest group, followed by replication, recombination and repair (1,453; 10.37%), transcription (1,310; 9.35%), signal transduction mechanisms (1,092; 7.79%), translation, ribosomal structure and biogenesis (1,017; 7.26%), posttranslational modification, protein turnover and chaperones (949; 6.77%), carbohydrate transport and metabolism (732; 5.22%), amino acid transport and metabolism (657; 4.69%), and energy production and conversion (583; 4.16%). However, only a few unigenes were assigned to cell motility and nuclear structure (18 and 2 unigenes, respectively). In addition, no unigene was assigned to extracellular structures. Furthermore, 417 (2.98%) unigenes were assigned to lipid transport and metabolism and 272 (1.94%) unigenes were assigned to coenzyme transport and metabolism (Figure 5). However, unigenes involved in terpenoid biosynthesis were found in the categories of lipid transport and metabolism and coenzyme transport and metabolism.

**Figure 6:**
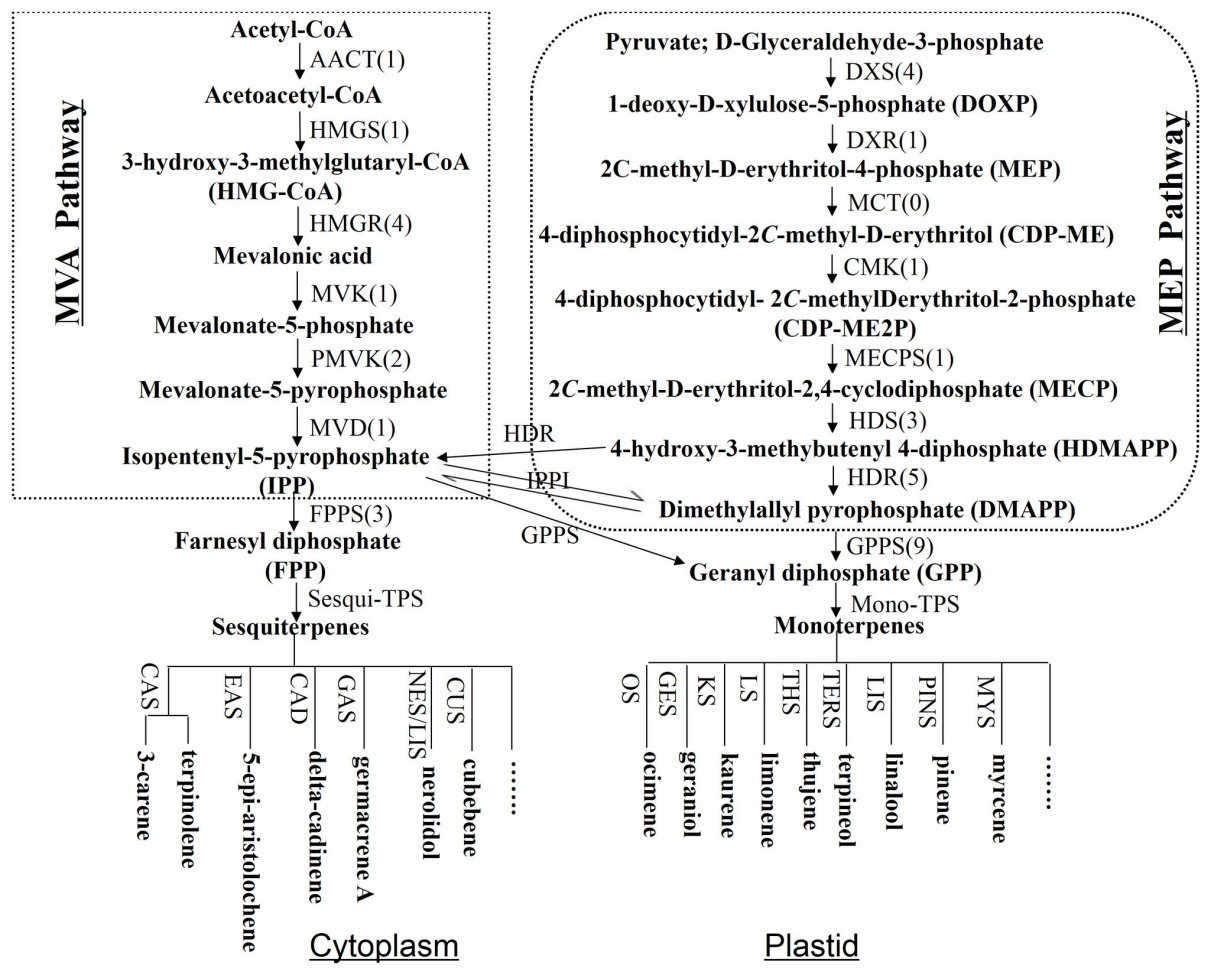
Monoterpenes and sesquiterpenes biosynthetic pathway in *L*.*cubeba* (adapted from Gahlan et al and Ma et al [57, 58]). Each enzyme name is followed in parentheses by the number of unigenes homologous to gene families encoding this enzyme. AACT: acetoacetyl-CoA thiolase; HMGS: 3-hydroxy-3-methylglutaryl-CoA synthase; HMGR: 3-hydroxy-3-methylglutaryl-CoA reductase; MVK: mevalonate kinase; PMVK: phosphomevalonate kinase; MVD: mevalonate diphosphate decarboxylase; DXS: 1-deoxy-dxylulose-5-phosphate synthase; DXR: 1-deoxy-D-xylulose-5-phosphate reductoisomerase; MCT: 4-diphosphocytidyl-2C-methyl-D- erythritol synthase; CMK: 4-diphosphocytidyl-2C-methyl-D-erythritol kinase; MECPS: 2C-methyl-D-erythritol 4-phosphate cytidylyltransferase; HDS: 1-hydroxy-2-methyl-2-(E)-butenyl-4-diphosphate synthase; HDR: 1-hydroxy-2-methyl-2-(E)-butenyl-4-diphosphate reductase; IPPI: isopentenyl-diphosphate isomerase; FPPS: farnesyl diphosphate synthase; GPPS: geranyl diphosphate synthase; Sesqui-TPS: Sesquiterpene synthase; Mono-TPS: Monoterpene synthase; CAS: (+)-3-carene synthase; CUS: beta-cubebene synthase; OS: Trans-ocimene synthase; GES: Geraniol synthase; KS: Ent-kaurene synthase; LS: limonene synthase; THS: Alpha-thujene synthase; TERS: Alpha-terpineol synthase; LIS: Linalool synthase; PINS: pinene synthase; EAS: 5-epi-aristolochene synthase; CAD: delta-cadinene synthase; GAS: Germacrene A synthase; NES/LIS: nerolidol/linalool synthase.

The KEGG database can be used to categorize gene functions with an emphasis on biochemical pathways. To better understand biological pathways in *L. cubeba*, a BLASTX search against the KEGG protein database was made on the assembled unigenes. Only 7,702 (11.2%) unigenes were assigned to 297 KEGG pathways (File S2). The predicted pathways represented the majority of plant biochemical pathways including metabolism, genetic information processing, cellular processes, and organism systems. The pathways with highest unigene representation were Chromosome (ko03036, 480 unigenes, 2.98%), followed by Ribosome (ko03011, 423 unigenes, 2.62%) and Spliceosome (ko03041, 409 unigenes, 2.54%). In our sequence dataset, 61 unigenes containing the MEP and MVA pathways were found to be potentially related to terpenoid backbone biosynthesis. At least one unigene from the transcriptome database of *L. cubeba* corresponded to the enzymes participating in the two pathways except 4-diphosphocytidyl-2C-methyl-D-erythritol synthase (MCT). The database contained more than one unigene each for 3-hydroxy-3-methylglutaryl-CoA reductase (HMGR), phosphomevalonate kinase (PMVK), farnesyl diphosphate synthase (FPPS), 1-deoxy-D-xylulose-5-phosphate synthase (DXS), 1-hydroxy-2-methyl-2-(E)-butenyl-4-diphosphate synthase (HDS), 1-hydroxy-2-methyl-2-(E)-butenyl- 4-diphosphate reductase (HDR) and geranyl diphosphate synthase (GPPS) (Figure 6). Thus, the large amount of transcriptomic information may accelerate the study of terpenoid biosynthesis in *L. cubeba*.

**Figure 7:**
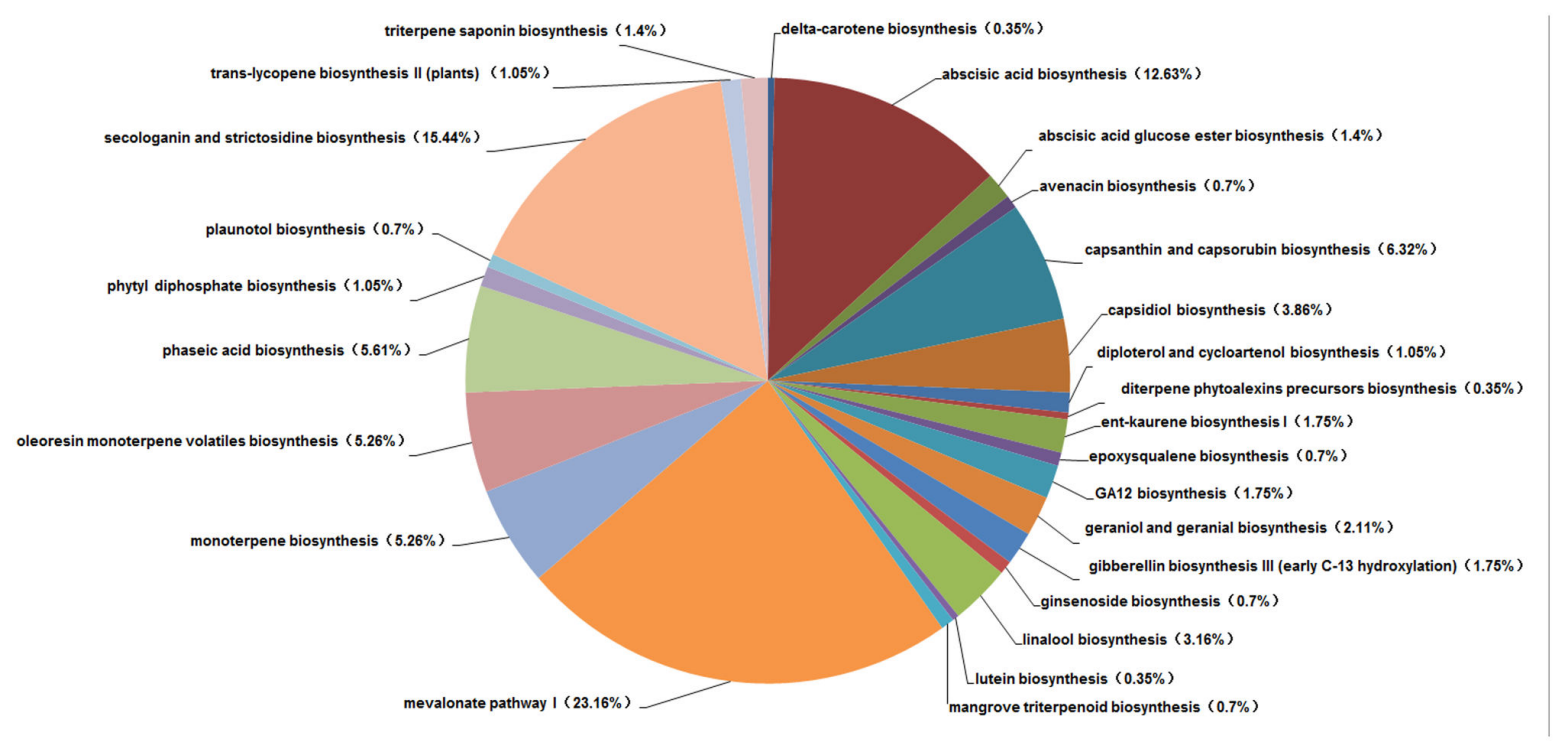
Terpenoid pathways represented in the PlantCyc annotation of the unigenes.

The PlantCyc database is a plant-specific metabolic pathway database, which includes experimentally supported, computationally predicted, and hypothetical pathways and enzymes. In the total unigenes (12,963) annotated using the PlantCyc database, we focused on terpenoid pathways (285 unigenes) to identify clues related to the medicinal properties of *L. cubeba* (Figure 7). A major share of the unigenes related to the terpenoid pathways were from mevalonate pathway I (23.16%) which provides the central intermediates for specific terpenoid biosynthesis, and 15.44% were involved in secologanin and strictosidine biosynthesis. Secologanin and strictosidine play important roles in pharmacology [24]. Meanwhile, the monoterpene biosynthesis pathways identified included linalool (3.16%), kaurene (1.75%), and geraniol and geranial (2.11%).

### SSR marker discovery

In total, 2,229 sequences containing 2,674 SSRs were identified from 10,270 unigenes, with 1,221 unigene sequences containing more than one SSR. Dinucleotide motifs and trinucleotide motifs were the most abundant, accounting for 50.90 and 46.07%, respectively (Table 3). The most abundant repeat type was AG/CT (1,210), followed by AAG/CTT (524), AGG/CCT (180), ATC/ATG (106), respectively. However, the number of tetra-, penta- and hexa-nucleotide motifs was less than 2% in the unigene sequences. No complex SSR motifs were detected, and not all unigenes had SSRs.

**Table 3:** Frequency of SSRs in *L. cubeba*.

Motif length	Repeat numbers							Total	%
	5	6	7	8	9	10	>10		
Di	-	419	270	202	166	198	106	1361	50.90
Tri	727	319	165	17	1	1	2	1232	46.07
Tetra	36	10	0	3	0	0	0	49	1.83
Penta	11	1	0	0	0	1	0	13	0.47
Hexa	8	4	5	0	2	0	0	19	0.71
Total	782	753	440	222	169	200	108	2674	
%	29.24	28.16	16.45	8.30	6.32	7.48	4.04		

### Phylogenetic analysis of TPS between L. *cubeba* and other plants

**Figure 8:**
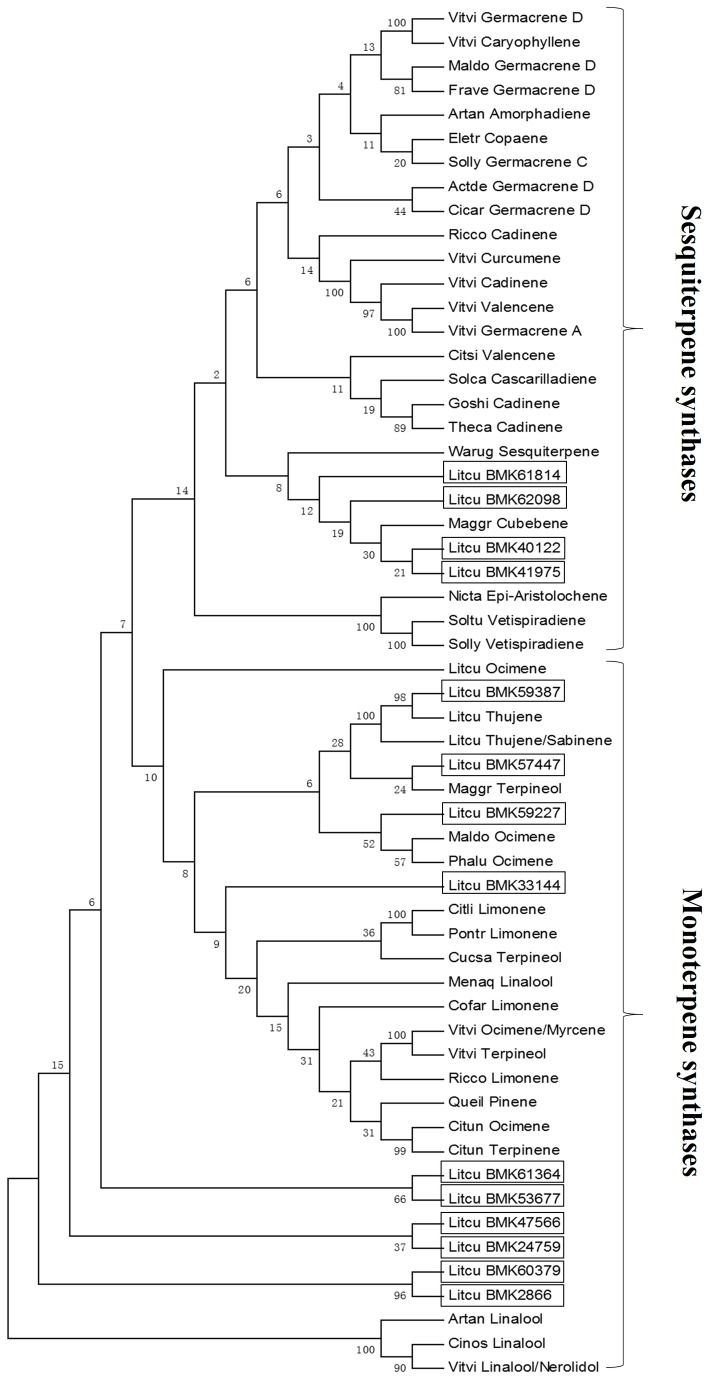
Phylogeny tree of terpene synthases. The two clades illustrate the likely enzymatic function of 14 *L*. *cubeba* unigenes. It also shows a distribution of the *L*. *cubeba* sequences throughout the two main clades of the tree. Vitvi Germacrene D (NP_001268213.1, (-)-germacrene D synthase, *Vitis* *vinifera*), Vitvi Caryophyllene (AEP17005.1, (E)-beta-caryophyllene synthase, *Vitis* *vinifera*), Maldo Germacrene D (AGB14625.1, germacrene-D synthase, *Malus* *domestica*), Frave Germacrene D (XP_004308410.1, (-)-germacrene D synthase-like, *Fragaria* *vesca* *subsp*. *vesca*), Artan Amorphadiene (AAF98444.1, amorpha-4,11-diene synthase, *Artemisia* *annua*), Eletr Copaene (ADK94034.1, alpha-copaene synthase, *Eleutherococcus* *trifoliatus*), Solly Germacrene C (XP_004242295.1, germacrene C synthase-like, *Solanum* *lycopersicum*), Actde Germacrene D (AAX16121.1, germacrene-D synthase, *Actinidia* *deliciosa*), Cicar Germacrene D (XP_004505471.1, (-)-germacrene D synthase-like, *Cicer* *arietinum*), Ricco Cadinene (XP_002523635.1, (+)-delta-cadinene synthase isozyme A, *Ricinus* *communis*), Vitvi Curcumene (ADR74200.1, beta-curcumene synthase, *Vitis* *vinifera*), Vitvi Cadinene (ADR74199.1, gamma-cadinene synthase, *Vitis* *vinifera*), Vitvi Valencene (NP_001268028.1, valencene synthase-like, *Vitis* *vinifera*), Vitvi Germacrene A (ADR66821.1, germacrene A synthase, *Vitis* *vinifera*), Citsi Valencene (AF441124_1, valencene synthase, *Citrus* *sinensis*), Solca Cascarilladiene (AAT72931.1, cascarilladiene synthase, *Solidago* *canadensis*), Goshi Cadinene (AAX44033.1, (+)-delta-cadinene synthase, *Gossypium* *hirsutum*), Theca Cadinene (EOY12645.1, delta-cadinene synthase isozyme A, *Theobroma* *cacao*), Warug Sesquiterpene (ACJ46047.1, sesquiterpene synthase, *Warburgia* *ugandensis*), Maggr Cubebene (B3TPQ6.1, beta-cubebene synthase, *Magnolia* *grandiflora*), Nicta Epi-Aristolochene (3M02.A, 5-Epi- Aristolochene Synthase, *Nicotiana* *tabacum*), Soltu Vetispiradiene (Q9XJ32.1, vetispiradiene synthase 1, *Solanum* *tuberosum*), Solly Vetispiradiene (AAG09949.1, AF171216_1, vetispiradiene synthase, *Solanum* *lycopersicum*), Litcu Ocimene (AEJ91554.1, trans-ocimene synthase, *Litsea* *cubeba*), Litcu Thujene (AEJ91555.1, alpha-thujene synthase, *Litsea* *cubeba*), Litcu Thujene/Sabinene (AEJ91556.1, alpha-thujene synthase/sabinene synthase, *Litsea* *cubeba*), Maggr Terpineol (ACC66282.1, alpha-terpineol synthase, *Magnolia* *grandiflora*), Maldo Ocimene (AGB14628.1, (E)-beta-ocimene synthase, *Malus* *domestica*), Phalu Ocimene (ABY65110.1, beta-ocimene synthase, *Phaseolus* *lunatus*), Citli Limonene (AAM53946.1|AF514289_1, (+)-limonene synthase 2, *Citrus* *limon*), Pontr Limonene (BAG74774.1, limonene synthase, *Poncirus* *trifoliata*), Cucsa Terpineol (XP_004161807.1, (-)-alpha-terpineol synthase-like, *Cucumis* *sativus*), Menaq Linalool (AAL99381.1, linalool synthase, *Mentha* *aquatica*), Cofar Limonene (CCM43927.1, limonene synthase, *Coffea* *arabica*), Vitvi Ocimene/Myrcene (ADR74206.1, (E)-beta-ocimene/myrcene synthase, *Vitis* *vinifera*), Vitvi Terpineol (NP_001268216.1, (-)-alpha-terpineol synthase, *Vitis* *vinifera*), Ricco Limonene (XP_002533355.1, (R)-limonene synthase, *Ricinus* *communis*), Queil Pinene (CAK55186.1, pinene synthase, *Quercus* *ilex*), Citun Ocimene (BAD91046.1, (E)-beta-ocimene synthase, *Citrus* *unshiu*), Citun Terpinene (BAD27259.1, gamma-terpinene synthase, *Citrus* *unshiu*), Artan Linalool (AAF13356.1|AF154124_1, (3R)-linalool synthase, *Artemisia* *annua*), Cinos Linalool (AFQ20811.1, S-(+)-linalool synthase, *Cinnamomum* *osmophloeum*), Vitvi Linalool/Nerolidol (ADR74212.1, (3S)-linalool/(E)-nerolidol synthase, *Vitis* *vinifera*).

To examine the phylogenetic relationship between the TPS domain proteins in *L. cubeba* and other plants, a phylogenetic tree was constructed from alignments of the TPS protein sequences (Figure 8). The phylogenetic tree was constructed using MEGA 4.0. Fourteen unique TPS sequences were identified from the *L. cubeba* unigenes (Figure 8). These sequences were divided into two clades involved in mono- and sesquiterpenoid biosynthesis. The phylogenetic analysis indicated that 10 unigenes (59387, 57447, 59227, 33144, 61364, 53677, 47566, 24759, 60379, and 2866) were likely to produce monoterpene backbones. The remaining four unigenes (61814, 62098, 40122, and 41975) were likely to be involved in sesquiterpene biosynthesis, and clustered with *Warburgia ugandensis* and *Magnolia grandiflora*.

### Genes related to the terpenoid biosynthesis pathway

**Figure 9:**
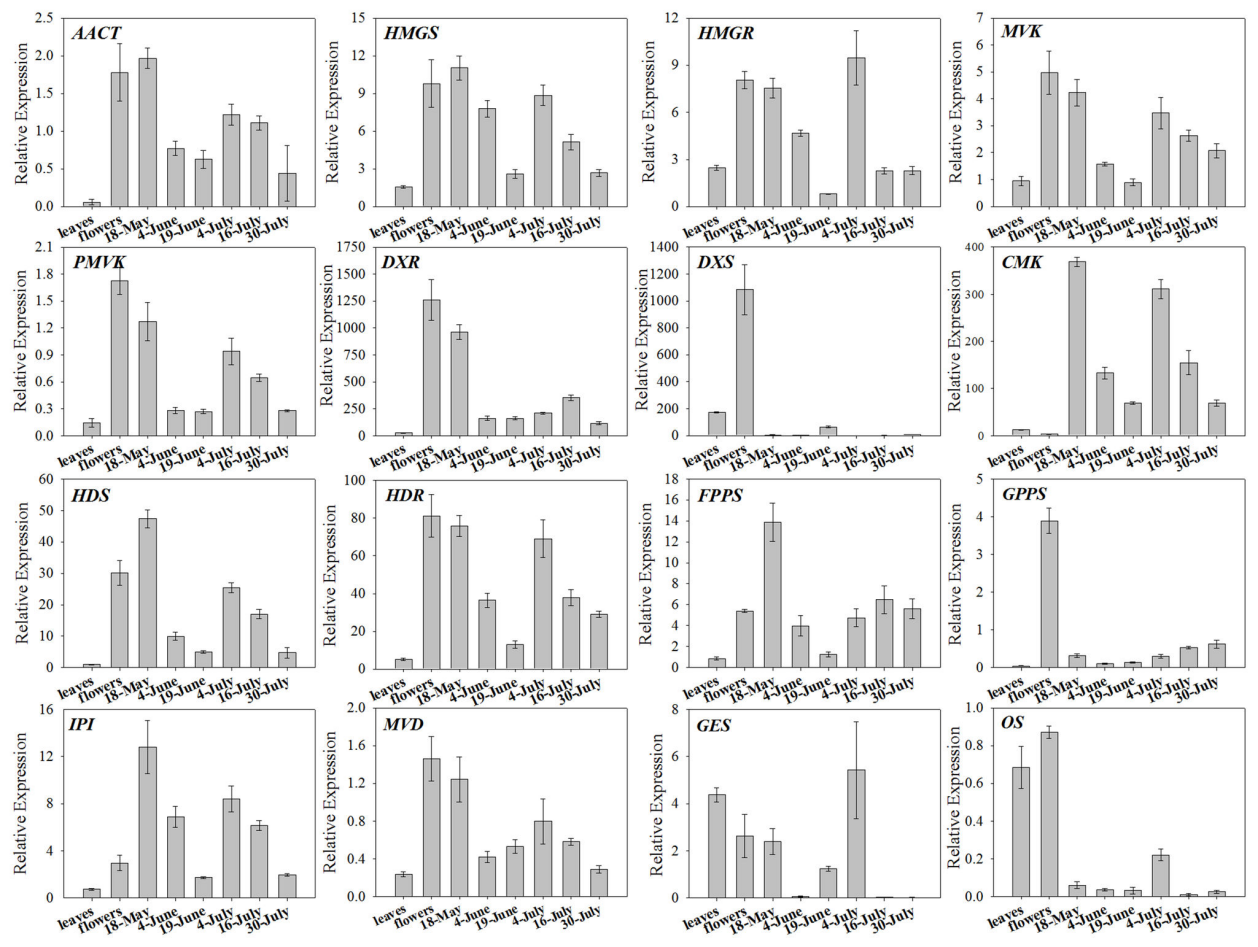
RT-qPCR analysis of 16 terpenoid pathway-related candidate unigenes in *L. cubeba.* The gene names, sequences and the primers used for RT-qPCR analysis are shown in File S3. Standard error of the mean for three biological replicates (nested with three technical replicates) is represented by the error bars.

The expression profiles were validated through RT-qPCR using 16 genes belonging to terpenoid biosynthetic pathways. All 16 candidates showed significantly differential expression levels in different pathways (Figure 9). The six genes of the MVA pathway (*AACT*, *HMGS*, *HMGR*, *MVK*, *PMVK* and *MVD*) showed the similar trends with their expressions levels being low in leaves but dramatically higher in flowers and young fruits by May 8^th^. The transcript levels decreased in the fruits on June 4^th^ and June 19^th^, and then up-regulated on July 4^th^. The five genes of the MEP pathway (*DXS*, *DXR*, *CMK*, *HDS* and *HDR*) showed a great change in the eight *L. cubeba* samples. In particular, *DXS* and *DXR* expression levels, which shared a similar profile, were high in flowers and lower in other samples. In addition, *FPPS* and *IPI* exhibited a similar trend with their highest expression levels occurring in young fruits. Geraniol synthase (*GES*), generating geraniol from geranyl diphosphate, was expressed at its highest level in developing fruits, while the expression analysis of trans-ocimene synthase (*OS*, generating ocimene from geranyl diphosphate) revealed higher mRNA levels in the leaf and flower tissues, and lower levels in the fruits.

## Discussion

Transcriptome sequencing has become an effective tool that is used to discover molecular markers and identify novel genes. In recent years, with the improvement of read length by paired-end sequencing, relatively short reads can be effectively assembled and have been successfully used for studying plants without genomic sequence [25,26]. Prior to this study, very few *L. cubeba* ESTs had been reported in the public databases and little sequence data were available. Here, we used RNA-Seq technology to profile the *L. cubeba* transcriptome on the Illumina HiSeqTM 2000 platform. A total of 6.66 Gbp of data with 23,460,490 high-quality reads were obtained from the platform, and 68,648 unigenes were identified by *de novo* assembly. Among them, about 56% were successfully annotated, indicating their relatively conserved functions. In addition, the functions of the unigenes were classified by COG, GO and the metabolic pathways. The non-annotated unigenes may represent poorly conserved regions [27], such as untranslated regions (UTRs), in *L. cubeba*. Furthermore, we estimated the gene or protein names and ORFs of the assembled unigenes, putative conserved domains, gene ontology terms, and potential metabolic pathways. Compared with previous transcriptomic studies using the same platform in other plants, such as *Chorispora bungeana* [28], *Carthamus tinctorius* [29], *Apium graveolens* [30] and *Boehmeria nivea* [31], the average length of *L. cubeba* unigenes was longer. To the best of our knowledge, this is the first attempt at the *de novo* sequencing and assembly (without a reference genome) of the *L. cubeba* transcriptome using Illumina paired-end sequencing technology. The results indicate that our final assembly quality is satisfactory. This work will provide a sequence source and facilitate further studies in gene cloning and functional analyses.

Predicting the number of genes and the potential functions of the unigenes are important parameters in the transcriptome sequencing projects. Due to the lack of a reference genome for *L. cubeba*, this information has been difficult to estimate the number of genes and predict the potential functions of the unigenes. Here, a large number of unigenes could be matched with unique known proteins in public databases, indicating that the Illumina sequencing project excavated a substantial fraction of gene resources from *L. cubeba*. However, 44% of the assembled unigenes did not match any known sequences in the public databases. These genes may perform specific roles in *L. cubeba* and be quite divergent from those of other plant species. *L. cubeba* has diverse medicinal and economic values, and belongs to the Lauraceae family, which lacks any published genomic resources. Thus, the transcriptome sequence generated in this study will be valuable for further research.

In this study, we presented significant sequence similarities of 48, 13 and 12% between *L. cubeba* and *V. vinifera*, *P. trichocarpa* and *R. communis* (Figure 3). According to the Angiosperm Phylogeny Group Ⅲ (APG Ⅲ), both *P. trichocarpa* and *R. communis* belong to the order Malpighiales in the Rosids family [32], while *V. vinifera* belongs to Vitalest, which is also classified in the Rosids. Thus, there is a close relationship between Malpighiales and Vitalest. Furthermore, reference genome sequences of *V. vinifera*, *P. trichocarpa* and *R. communis* are available in the public databases [33-35]. Therefore, sequence similarities are high between *L. cubeba* and the three plant species due to their genome sequences. Here, *L. cubeba* and *V. vinifera* showed a close relationship at the molecular level. However, the evidence appears to contradict the taxonomic result. This discrepancy deserves further study.

The number of terpene synthases identified from *L. cubeba*, ten monoterpene synthases and four sesquiterpene synthases, and the three functionally characterized monoterpene synthase gene sequences have been deposited in the NCBI GenBank database and functionally characterized [11]. At present, a relatively large number of TPSs have been found in plants with genomic sequences. Thirty-two genes encoding putatively active mono-, sesqui-, and di-TPSs were reported in the *Arabidopsis* genome [36], 69 putatively functional genes in the genome of *V. vinifera* [37], 44 genes in the cultivated tomato (*Solanum lycopersicum*) genome [38], and 55 putative genes in cultivated apple (*Malus domestica*) genome [39]. Whereas, a relatively small number of TPSs were identified in plants without genomic sequences. For example, 17 unique terpene synthase sequences were identified in the *Thapsia laciniata* transcriptome [40]. The terpene synthases found in *L. cubeba* were mono- and sesquiterpene synthases, which corresponds well to the dominant components in its essential oils. Additionally, sequence comparisons revealed that all the TPSs had similar properties with respect to their native molecular masses [41]. Many TPSs catalyze the formation of a single product, and some TPSs catalyze the formation of multiple products [42]. The mono-TPS and sesqui-TPS described here are necessary to undergo biochemical characterization.

At present, the terpenoid biosynthetic pathway has been well studied and many genes have been identified that participate in this pathway in other plants [39,43,44]. According to our KEGG pathway enrichment results, 61 unigenes were found to be involved in terpenoid backbone biosynthesis. In this study, many unigene sequences assembled were not the full-length cDNAs and the protein sequences predicted were incomplete. Thus, MCT or gene families involved in terpenoid biosynthesis were not found using the transcriptome results. An analysis of the expression pattern of 16 genes related to terpenoid biosynthesis showed that there are 12 genes with high expression levels in the flowers. *DXS*, *GPPS* and *OS* were hardly detected in the different developing stages of fruits. The expression profile of *OS* was consistent with the previous results [11], which showed its expression mainly in leaf tissue. Therefore, the identification of these genes will improve our understanding of the mechanisms of terpenoid biosynthesis. This study indicates that Illumina sequencing technology can be applied as a rapid method for *de novo* transcriptome analysis of non-model plants with unavailable genomic information. We believe that the transcriptome dataset will accelerate research on the gene expression and functional genomics of *L. cubeba*.

## Materials and Methods

### Plant materials

All samples of *L. cubeba* were collected from Fuyang’s Urban Forest Park, Zhejiang Province. No specific permits were required from the park to select samples. The park is not privately-owned and the field studies did not involve protected species. The following plant tissues were sampled: flower buds, full open flowers, young leaves, leaf buds and fruits of seven different ripening stages, 45 days after flowering (DAF) (5/3/2012), 60 DAF (5/18/2012), 77 DAF (6/4/2012), 92 DAF (6/19/2012), 106 DAF (7/4/2012), 118 DAF (7/16/2012), and 132 DAF (7/30/2012). Each tissue was harvested from five different plants. All samples were immediately frozen in liquid nitrogen and stored at −80°C for later use.

### RNA extraction, library construction and RNA-seq

Total RNA from each sample was isolated separately using the RN38 EASYspin plus Plant RNA kit (Aidlab Biotech, Beijing, China). The purified RNA concentration was quantified with a spectrophotometer (UV-Vis Spectrophotometer, Quawell Q5000, San Jose, CA, USA), and RNA integrity was evaluated using an Agilent 2100 Bioanalyzer (Agilent Technologies, Santa Clara, CA, USA). Samples of 2.0 µg high-quality RNA from each sample were combined into a single large pool in an attempt to maximize the diversity of transcriptional units.

The mRNA-seq library was constructed using Illumina’s TruSeq RNA Sample Preparation Kit (Illumina Inc, San Diego, CA, USA). The isolation of mRNA, fragment interruption, cDNA synthesis, addition of adapters, PCR amplification and RNA-Seq were performed by staff at Beijing BioMarker Technologies (Beijing, China). Poly-A mRNA was isolated using poly-T oligo-attached magnetic beads, and then broken into small pieces using divalent cations under an elevated temperature. The cleaved RNA fragments were copied into first strand cDNA using reverse transcriptase and random primers. This was followed by second strand cDNA synthesis using DNA Polymerase I and RNase H. A single ‘A’ base was ligated to the short fragments after being purified using a MinElute PCR Purification Kit (Qiagen, Dusseldorf, Germany), preparing them for ligation to the sequencing adapters. Fragments (200 bp ± 25 bp) were then separated by agarose gel electrophoresis and selected for PCR amplification as sequencing templates. Finally, the mRNA-seq library was constructed for sequencing on the Illumina HiSeq^TM^ 2000 sequencing platform.

### Sequence data analysis and assembly

To obtain high-quality clean read data for *de novo* assembly, the raw reads from mRNA-seq were filtered by discarding the reads with adaptor contamination, masking low-quality reads with ambiguous ‘N’ bases and removing the reads in which more than 10% bases had a *Q*-value <20 [20,45]. The clean reads were assembled into contigs using the Trinity method (http://trinityrnaseq.sourceforge.net/), which is efficient in reconstructing full-length transcripts across a broad range of expression levels and sequencing depths [46]. We used the Trinity method with an optimized k-mer length of 25 for *de novo* assembly. Subsequently, the contigs were linked into transcripts according to the paired-end information of the sequences. Then the transcripts were clustered based on nucleotide sequence identity. The longest transcripts in the cluster units were regarded as unigenes to eliminate redundant sequences, and then the unigenes were combined to produce the final assembly used for annotation.

### Functional annotation

To determine the functional annotation of the unigenes, a BLASTX search was performed against the NCBI Nr database with an E-value≤10^-5^ and other databases, including SwissProt, Protein Information Resource (PIR), Protein Research Foundation (PRF) and Protein Data Bank (PDB). Gene names were assigned based on the annotation of the closest UniProt match, with uninformative descriptions excluded. A BLASTN search was also performed against the NCBI Nt database, GenBank, RefSeq and PDB using a protein query. The ORFs were identified as the nucleotide sequence or as the protein translation provided by the “GetORF” program from the EMBOSS software package [47]. The longest ORF was extracted for each unigene. The expression abundance of the unigenes was represented by the number of reads per kilobase of exon model per million mapped reads (RPKM) [48]. The RPKM method is able to reflect the molar concentration of a transcript by normalizing for RNA length and for the total read number. The Blast2GO program was used to assign GO terms with an E-value≤10^-5^ including molecular functions, biological processes, and cellular components [49,50]. Annotations of *L. cubeba* unigenes were then used to predict biochemical pathways using the Pathway Tools [51]. KEGG pathways were retrieved from KEGG web server (http://www.genome.jp/kegg/) [52]. The output of the KEGG analysis includes KO assignments and KEGG pathways that are populated with the KO assignments. The metabolic pathways were annotated by PlantCyc Enzymes database v7.0 (http://www.plantcyc.org), and the terpenoid pathways were highlighted in the PlantCyc annotation [53,54].

### Detection of SSR markers

The assembled sequences longer than 1kb were used for the detection of SSR markers. Potential SSR markers were detected among the 10,270 unigenes using MISA software (http://pgrc.ipk-gatersleben.de/misa/). The parameters were designed for the identification of perfect dinucleotide motifs with a minimum of six repeats, and tri-, tetra-, penta-, and hexanucleotide motifs with a minimum of five repeats [26,55].

### Alignment and phylogenetic tree building

A Hidden Markov Model (HMM) was employed to identify the terpene synthase genes of the *L. cubeba* transcriptome. The profile of the terpene synthase N-terminal domain (pfam01397) used for the HMM search (HMMER 3.1, http://hmmer.janelia.org/) was downloaded from the Pfam database (http://pfam.sanger.ac.uk/). A total of 14 terpene synthase sequences were obtained with an E-value threshold of 0.1. The phylogenetic tree was created by MEGA4.0 neighbor-joining software with 1000 bootstrap trials.

### Real-time quantitative PCR analysis (RT-qPCR)

The *L. cubeba* transcriptome database was mined for genes involved in terpenoid biosynthetic pathways. The expression profiles include the genes involved in the MVA pathway (*AACT*, *HMGS*, *HMGR*, *MVK*, *PMVK* and *MVD*), MEP pathway (*DXS*, *DXR*, *CMK*, *HDS* and *HDR*) and other genes (*IPPI*, *FPPS*, *GPPS*, *GES* and *OS*). cDNA synthesis was performed with 3 µg total RNA from leaves, flowers and six developmental fruit stages (May 18^th^, June 4^th^, June 19^th^, July 4^th^, July 16^th^, July 30^th^) using the Superscript Ш First Strand Synthesis system followed by the RNase H step (Invitrogen, Carlsbad, USA), according to the manufacturer’s protocol in a total volume of 20 µl. Primer pairs were designed using Primer3 (http://frodo.wi.mit.edu/primer3/) with the following parameters: Tm of approximately 60°C, product size range of 120-200 base pairs, primer sequences with a length of 20 nucleotides, and a GC content of 45 to 55%. The gene names, sequences and the primers used for RT-qPCR analysis are listed in File S3. To quantify the expression level the selected genes, the *L. cubeba UBC* (ubiquitin-conjugating enzyme E2) gene was used as an endogenous control. RT-qPCR reactions were performed in 96-well plates using a 7300 Real Time PCR System (Applied Biosystems, CA, USA) and a SYBR^®^ Premix Ex Taq™ Kit (TaKaRa, Dalian, China). PCR reactions were prepared in 20 µl volumes containing 2 µl of 30-fold diluted synthesized cDNA, 10 µl 2×SYBR^®^ Premix Ex Taq^TM^, 0.4 µl 10 µM forward primer, 0.4 µl 10 µM reverse primer, 0.4 µl 50× RO× reference dye and 6.8 µl sterile distilled water. The cycling conditions were recommended by the manufacturer (30 s at 95°C, 40 cycles of 95°C for 5 s, and 60°C for 31 s). The specificity of the amplicons was verified by melting curve analysis (60 to 95°C) after 40 PCR cycles. Three biological replicates (nested with three technical replicates) per sample were carried out, and the data analysis was performed as described by Pfaffl [56].

## Supporting Information

File S1 **Summary of Illumina transcriptome sequencing for *L. cubeba.***
(DOC)

File S2 **Pathway assignment based on KEGG.**
(DOC)

File S3 **The gene names, sequences and the primers used for RT-qPCR analysis.**
(DOC)
