# Supplementary material for: Transcriptome Sequencing and Expression Analysis of Terpenoid Biosynthesis Genes in Litsea cubeba
Source: PLoS One. 2013 Oct 9;8(10):e76890. doi: 10.1371/journal.pone.0076890 (PMC3793921; doi:10.1371/journal.pone.0076890)
Supplement: File S1 — Summary of Illumina transcriptome sequencing for L. cubeba. (DOC) [file pone.0076890.s001.doc]

Table S1 Summary of Illumina transcriptome sequencing for *L. cubeba*

| Sample | Raw reads | Total nucleotides(bp) | Cleaning reads | Q20 (%) | GC (%) |
| --- | --- | --- | --- | --- | --- |
| *L. cubeba* | 36,618,326 | 6,661,865,601 | 23,460,490 | 100 | 47.66 |
