# Supplementary material for: Transcriptome Sequencing and Expression Analysis of Terpenoid Biosynthesis Genes in Litsea cubeba
Source: PLoS One. 2013 Oct 9;8(10):e76890. doi: 10.1371/journal.pone.0076890 (PMC3793921; doi:10.1371/journal.pone.0076890)
Supplement: File S2 — Pathway assignment based on KEGG. (DOC) [file pone.0076890.s002.doc]

File S2 Pathway assignment based on KEGG

|  | Pathway | Pathway ID | Count (16,130) | Percentage |
| --- | --- | --- | --- | --- |
| 1 | Chromosome | ko03036 | 480 | 0.029758215 |
| 2 | Ribosome | ko03011 | 423 | 0.026224427 |
| 3 | Spliceosome | ko03041 | 409 | 0.025356479 |
| 4 | Ubiquitin system | ko04121 | 399 | 0.024736516 |
| 5 | DNA repair and recombination proteins | ko03400 | 337 | 0.020892746 |
| 6 | Chaperones and folding catalysts | ko03110 | 315 | 0.019528828 |
| 7 | Peptidases | ko01002 | 305 | 0.018908865 |
| 8 | Glycosyltransferases | ko01003 | 266 | 0.016491011 |
| 9 | RNA transport | ko03013 | 266 | 0.016491011 |
| 10 | Oxidative phosphorylation | ko00190 | 251 | 0.015561066 |
| 11 | Plant hormone signal transduction | ko04075 | 242 | 0.0150031 |
| 12 | Transcription factors | ko03000 | 234 | 0.01450713 |
| 13 | Purine metabolism | ko00230 | 227 | 0.014073156 |
| 14 | DNA replication proteins | ko03032 | 221 | 0.013701178 |
| 15 | Protein kinases | ko01001 | 221 | 0.013701178 |
| 16 | Starch and sucrose metabolism | ko00500 | 201 | 0.012461252 |
| 17 | Ubiquitin mediated proteolysis | ko04120 | 192 | 0.011903286 |
| 18 | Huntington's disease | ko05016 | 191 | 0.01184129 |
| 19 | Glycolysis / Gluconeogenesis | ko00010 | 189 | 0.011717297 |
| 20 | Cell cycle | ko04110 | 178 | 0.011035338 |
| 21 | Amino sugar and nucleotide sugar metabolism | ko00520 | 170 | 0.010539368 |
| 22 | Pyrimidine metabolism | ko00240 | 168 | 0.010415375 |
| 23 | Cell cycle - yeast | ko04111 | 163 | 0.010105394 |
| 24 | Plant-pathogen interaction | ko04626 | 162 | 0.010043397 |
| 25 | mRNA surveillance pathway | ko03015 | 154 | 0.009547427 |
| 26 | Alzheimer's disease | ko05010 | 152 | 0.009423435 |
| 27 | Ribosome biogenesis in eukaryotes | ko03008 | 152 | 0.009423435 |
| 28 | Translation factors | ko03012 | 150 | 0.009299442 |
| 29 | Methane metabolism | ko00680 | 148 | 0.009175449 |
| 30 | Parkinson's disease | ko05012 | 146 | 0.009051457 |
| 31 | Endocytosis | ko04144 | 142 | 0.008803472 |
| 32 | Photosynthesis proteins | ko00194 | 142 | 0.008803472 |
| 33 | RNA degradation | ko03018 | 141 | 0.008741476 |
| 34 | Proteasome | ko03051 | 138 | 0.008555487 |
| 35 | Cytoskeleton proteins | ko04812 | 134 | 0.008307502 |
| 36 | Lipid biosynthesis proteins | ko01004 | 131 | 0.008121513 |
| 37 | Peroxisome | ko04146 | 130 | 0.008059516 |
| 38 | Carbon fixation in photosynthetic organisms | ko00710 | 129 | 0.00799752 |
| 39 | Oocyte meiosis | ko04114 | 122 | 0.007563546 |
| 40 | Pyruvate metabolism | ko00620 | 115 | 0.007129572 |
| 41 | Meiosis - yeast | ko04113 | 113 | 0.00700558 |
| 42 | Glycerophospholipid metabolism | ko00564 | 112 | 0.006943583 |
| 43 | Amino acid related enzymes | ko01007 | 109 | 0.006757595 |
| 44 | Photosynthesis | ko00195 | 108 | 0.006695598 |
| 45 | Phagosome | ko04145 | 103 | 0.006385617 |
| 46 | Lysosome | ko04142 | 100 | 0.006199628 |
| 47 | Glutathione metabolism | ko00480 | 98 | 0.006075635 |
| 48 | Phenylpropanoid biosynthesis | ko00940 | 95 | 0.005889647 |
| 49 | Antigen processing and presentation | ko04612 | 94 | 0.00582765 |
| 50 | Pathways in cancer | ko05200 | 93 | 0.005765654 |
| 51 | Cysteine and methionine metabolism | ko00270 | 92 | 0.005703658 |
| 52 | Pentose phosphate pathway | ko00030 | 92 | 0.005703658 |
| 53 | Citrate cycle (TCA cycle) | ko00020 | 89 | 0.005517669 |
| 54 | Phenylalanine metabolism | ko00360 | 89 | 0.005517669 |
| 55 | Nucleotide excision repair | ko03420 | 88 | 0.005455673 |
| 56 | Arginine and proline metabolism | ko00330 | 87 | 0.005393676 |
| 57 | DNA replication | ko03030 | 87 | 0.005393676 |
| 58 | Fructose and mannose metabolism | ko00051 | 86 | 0.00533168 |
| 59 | Alanine, aspartate and glutamate metabolism | ko00250 | 85 | 0.005269684 |
| 60 | Insulin signaling pathway | ko04910 | 79 | 0.004897706 |
| 61 | Nitrogen metabolism | ko00910 | 77 | 0.004773714 |
| 62 | Glycerolipid metabolism | ko00561 | 76 | 0.004711717 |
| 63 | Homologous recombination | ko03440 | 75 | 0.004649721 |
| 64 | Toxoplasmosis | ko05145 | 75 | 0.004649721 |
| 65 | Progesterone-mediated oocyte maturation | ko04914 | 74 | 0.004587725 |
| 66 | Wnt signaling pathway | ko04310 | 73 | 0.004525728 |
| 67 | Aminoacyl-tRNA biosynthesis | ko00970 | 72 | 0.004463732 |
| 68 | Inositol phosphate metabolism | ko00562 | 72 | 0.004463732 |
| 69 | Mismatch repair | ko03430 | 69 | 0.004277743 |
| 70 | N-Glycan biosynthesis | ko00510 | 68 | 0.004215747 |
| 71 | Regulation of actin cytoskeleton | ko04810 | 67 | 0.004153751 |
| 72 | Glyoxylate and dicarboxylate metabolism | ko00630 | 65 | 0.004029758 |
| 73 | Pentose and glucuronate interconversions | ko00040 | 65 | 0.004029758 |
| 74 | Fatty acid metabolism | ko00071 | 64 | 0.003967762 |
| 75 | Fc gamma R-mediated phagocytosis | ko04666 | 64 | 0.003967762 |
| 76 | RNA polymerase | ko03020 | 62 | 0.003843769 |
| 77 | Terpenoid backbone biosynthesis | ko00900 | 61 | 0.003781773 |
| 78 | MAPK signaling pathway | ko04010 | 60 | 0.003719777 |
| 79 | Phosphatidylinositol signaling system | ko04070 | 60 | 0.003719777 |
| 80 | Fatty acid biosynthesis | ko00061 | 57 | 0.003533788 |
| 81 | Measles | ko05162 | 57 | 0.003533788 |
| 82 | Porphyrin and chlorophyll metabolism | ko00860 | 57 | 0.003533788 |
| 83 | Propanoate metabolism | ko00640 | 57 | 0.003533788 |
| 84 | Valine, leucine and isoleucine degradation | ko00280 | 57 | 0.003533788 |
| 85 | PPAR signaling pathway | ko03320 | 56 | 0.003471792 |
| 86 | Tyrosine metabolism | ko00350 | 55 | 0.003409795 |
| 87 | Biosynthesis of unsaturated fatty acids | ko01040 | 54 | 0.003347799 |
| 88 | Tryptophan metabolism | ko00380 | 54 | 0.003347799 |
| 89 | Galactose metabolism | ko00052 | 53 | 0.003285803 |
| 90 | Transporters | ko02000 | 52 | 0.003223807 |
| 91 | Base excision repair | ko03410 | 51 | 0.00316181 |
| 92 | Vibrio cholerae infection | ko05110 | 51 | 0.00316181 |
| 93 | beta-Alanine metabolism | ko00410 | 51 | 0.00316181 |
| 94 | Basal transcription factors | ko03022 | 50 | 0.003099814 |
| 95 | Glycine, serine and threonine metabolism | ko00260 | 50 | 0.003099814 |
| 96 | Phenylalanine, tyrosine and tryptophan biosynthesis | ko00400 | 49 | 0.003037818 |
| 97 | Prenyltransferases | ko01006 | 49 | 0.003037818 |
| 98 | p53 signaling pathway | ko04115 | 48 | 0.002975821 |
| 99 | Protein export | ko03060 | 47 | 0.002913825 |
| 100 | Drug metabolism - cytochrome P450 | ko00982 | 46 | 0.002851829 |
| 101 | Metabolism of xenobiotics by cytochrome P450 | ko00980 | 46 | 0.002851829 |
| 102 | Systemic lupus erythematosus | ko05322 | 46 | 0.002851829 |
| 103 | Cardiac muscle contraction | ko04260 | 44 | 0.002727836 |
| 104 | TGF-beta signaling pathway | ko04350 | 44 | 0.002727836 |
| 105 | Valine, leucine and isoleucine biosynthesis | ko00290 | 44 | 0.002727836 |
| 106 | Rheumatoid arthritis | ko05323 | 43 | 0.00266584 |
| 107 | Amyotrophic lateral sclerosis (ALS) | ko05014 | 42 | 0.002603844 |
| 108 | Ascorbate and aldarate metabolism | ko00053 | 42 | 0.002603844 |
| 109 | Carbon fixation pathways in prokaryotes | ko00720 | 42 | 0.002603844 |
| 110 | Carotenoid biosynthesis | ko00906 | 42 | 0.002603844 |
| 111 | Prostate cancer | ko05215 | 42 | 0.002603844 |
| 112 | Ether lipid metabolism | ko00565 | 41 | 0.002541847 |
| 113 | Various types of N-glycan biosynthesis | ko00513 | 41 | 0.002541847 |
| 114 | Butanoate metabolism | ko00650 | 39 | 0.002417855 |
| 115 | Epithelial cell signaling in Helicobacter pylori infection | ko05120 | 39 | 0.002417855 |
| 116 | GTP-binding proteins | ko04031 | 39 | 0.002417855 |
| 117 | Neurotrophin signaling pathway | ko04722 | 37 | 0.002293862 |
| 118 | Tight junction | ko04530 | 37 | 0.002293862 |
| 119 | Colorectal cancer | ko05210 | 36 | 0.002231866 |
| 120 | Cytochrome P450 | ko00199 | 36 | 0.002231866 |
| 121 | Secretion system | ko02044 | 36 | 0.002231866 |
| 122 | mTOR signaling pathway | ko04150 | 36 | 0.002231866 |
| 123 | Chagas disease (American trypanosomiasis) | ko05142 | 35 | 0.00216987 |
| 124 | Pathogenic Escherichia coli infection | ko05130 | 34 | 0.002107874 |
| 125 | Collecting duct acid secretion | ko04966 | 33 | 0.002045877 |
| 126 | Cyanoamino acid metabolism | ko00460 | 33 | 0.002045877 |
| 127 | Glycosylphosphatidylinositol(GPI)-anchor biosynthesis | ko00563 | 33 | 0.002045877 |
| 128 | GnRH signaling pathway | ko04912 | 33 | 0.002045877 |
| 129 | Photosynthesis - antenna proteins | ko00196 | 33 | 0.002045877 |
| 130 | Bacterial invasion of epithelial cells | ko05100 | 32 | 0.001983881 |
| 131 | Shigellosis | ko05131 | 32 | 0.001983881 |
| 132 | Gap junction | ko04540 | 31 | 0.001921885 |
| 133 | NOD-like receptor signaling pathway | ko04621 | 31 | 0.001921885 |
| 134 | Sulfur metabolism | ko00920 | 31 | 0.001921885 |
| 135 | Ubiquinone and other terpenoid-quinone biosynthesis | ko00130 | 31 | 0.001921885 |
| 136 | Calcium signaling pathway | ko04020 | 30 | 0.001859888 |
| 137 | Chloroalkane and chloroalkene degradation | ko00625 | 30 | 0.001859888 |
| 138 | Hepatitis C | ko05160 | 30 | 0.001859888 |
| 139 | Long-term potentiation | ko04720 | 30 | 0.001859888 |
| 140 | alpha-Linolenic acid metabolism | ko00592 | 30 | 0.001859888 |
| 141 | Selenocompound metabolism | ko00450 | 29 | 0.001797892 |
| 142 | Type II diabetes mellitus | ko04930 | 28 | 0.001735896 |
| 143 | Adipocytokine signaling pathway | ko04920 | 27 | 0.0016739 |
| 144 | Cytosolic DNA-sensing pathway | ko04623 | 27 | 0.0016739 |
| 145 | Histidine metabolism | ko00340 | 27 | 0.0016739 |
| 146 | Lysine degradation | ko00310 | 27 | 0.0016739 |
| 147 | Prion diseases | ko05020 | 27 | 0.0016739 |
| 148 | Regulation of autophagy | ko04140 | 27 | 0.0016739 |
| 149 | SNAREs | ko04131 | 27 | 0.0016739 |
| 150 | Sphingolipid metabolism | ko00600 | 27 | 0.0016739 |
| 151 | Circadian rhythm - mammal | ko04710 | 25 | 0.001549907 |
| 152 | Flavonoid biosynthesis | ko00941 | 25 | 0.001549907 |
| 153 | Focal adhesion | ko04510 | 25 | 0.001549907 |
| 154 | Renal cell carcinoma | ko05211 | 25 | 0.001549907 |
| 155 | Steroid biosynthesis | ko00100 | 25 | 0.001549907 |
| 156 | Viral myocarditis | ko05416 | 25 | 0.001549907 |
| 157 | Isoquinoline alkaloid biosynthesis | ko00950 | 24 | 0.001487911 |
| 158 | Axon guidance | ko04360 | 23 | 0.001425914 |
| 159 | Taurine and hypotaurine metabolism | ko00430 | 23 | 0.001425914 |
| 160 | ABC transporters | ko02010 | 22 | 0.001363918 |
| 161 | Adherens junction | ko04520 | 22 | 0.001363918 |
| 162 | Arachidonic acid metabolism | ko00590 | 22 | 0.001363918 |
| 163 | Cellular antigens | ko04090 | 22 | 0.001363918 |
| 164 | Drug metabolism - other enzymes | ko00983 | 22 | 0.001363918 |
| 165 | Folate biosynthesis | ko00790 | 22 | 0.001363918 |
| 166 | SNARE interactions in vesicular transport | ko04130 | 22 | 0.001363918 |
| 167 | Vascular smooth muscle contraction | ko04270 | 22 | 0.001363918 |
| 168 | Melanogenesis | ko04916 | 21 | 0.001301922 |
| 169 | One carbon pool by folate | ko00670 | 21 | 0.001301922 |
| 170 | Pantothenate and CoA biosynthesis | ko00770 | 21 | 0.001301922 |
| 171 | Protein digestion and absorption | ko04974 | 21 | 0.001301922 |
| 172 | VEGF signaling pathway | ko04370 | 21 | 0.001301922 |
| 173 | Glycan bindng proteins | ko04091 | 20 | 0.001239926 |
| 174 | Long-term depression | ko04730 | 20 | 0.001239926 |
| 175 | Notch signaling pathway | ko04330 | 20 | 0.001239926 |
| 176 | Two-component system | ko02020 | 20 | 0.001239926 |
| 177 | Mineral absorption | ko04978 | 19 | 0.001177929 |
| 178 | Retinol metabolism | ko00830 | 19 | 0.001177929 |
| 179 | Streptomycin biosynthesis | ko00521 | 19 | 0.001177929 |
| 180 | Endometrial cancer | ko05213 | 18 | 0.001115933 |
| 181 | Glioma | ko05214 | 18 | 0.001115933 |
| 182 | MAPK signaling pathway - yeast | ko04011 | 18 | 0.001115933 |
| 183 | Natural killer cell mediated cytotoxicity | ko04650 | 18 | 0.001115933 |
| 184 | Nicotinate and nicotinamide metabolism | ko00760 | 18 | 0.001115933 |
| 185 | Pancreatic cancer | ko05212 | 18 | 0.001115933 |
| 186 | B cell receptor signaling pathway | ko04662 | 17 | 0.001053937 |
| 187 | Bile secretion | ko04976 | 17 | 0.001053937 |
| 188 | Cell cycle - Caulobacter | ko04112 | 17 | 0.001053937 |
| 189 | Lysine biosynthesis | ko00300 | 17 | 0.001053937 |
| 190 | Naphthalene degradation | ko00626 | 17 | 0.001053937 |
| 191 | Zeatin biosynthesis | ko00908 | 17 | 0.001053937 |
| 192 | Non-homologous end-joining | ko03450 | 16 | 0.00099194 |
| 193 | Renin-angiotensin system | ko04614 | 16 | 0.00099194 |
| 194 | Sulfur relay system | ko04122 | 16 | 0.00099194 |
| 195 | Apoptosis | ko04210 | 15 | 0.000929944 |
| 196 | Bacterial secretion system | ko03070 | 15 | 0.000929944 |
| 197 | Chemokine signaling pathway | ko04062 | 15 | 0.000929944 |
| 198 | Fc epsilon RI signaling pathway | ko04664 | 15 | 0.000929944 |
| 199 | Non-small cell lung cancer | ko05223 | 15 | 0.000929944 |
| 200 | Glycosaminoglycan degradation | ko00531 | 14 | 0.000867948 |
| 201 | Ion channels | ko04040 | 14 | 0.000867948 |
| 202 | T cell receptor signaling pathway | ko04660 | 14 | 0.000867948 |
| 203 | Thyroid cancer | ko05216 | 14 | 0.000867948 |
| 204 | Chronic myeloid leukemia | ko05220 | 13 | 0.000805952 |
| 205 | Limonene and pinene degradation | ko00903 | 13 | 0.000805952 |
| 206 | Osteoclast differentiation | ko04380 | 13 | 0.000805952 |
| 207 | Toll-like receptor signaling pathway | ko04620 | 13 | 0.000805952 |
| 208 | Circadian rhythm - plant | ko04712 | 12 | 0.000743955 |
| 209 | Diterpenoid biosynthesis | ko00904 | 12 | 0.000743955 |
| 210 | ErbB signaling pathway | ko04012 | 12 | 0.000743955 |
| 211 | Leishmaniasis | ko05140 | 12 | 0.000743955 |
| 212 | Linoleic acid metabolism | ko00591 | 12 | 0.000743955 |
| 213 | Melanoma | ko05218 | 12 | 0.000743955 |
| 214 | Riboflavin metabolism | ko00740 | 12 | 0.000743955 |
| 215 | Stilbenoid, diarylheptanoid and gingerol biosynthesis | ko00945 | 12 | 0.000743955 |
| 216 | Tropane, piperidine and pyridine alkaloid biosynthesis | ko00960 | 12 | 0.000743955 |
| 217 | Acute myeloid leukemia | ko05221 | 11 | 0.000681959 |
| 218 | Aldosterone-regulated sodium reabsorption | ko04960 | 11 | 0.000681959 |
| 219 | Aminobenzoate degradation | ko00627 | 11 | 0.000681959 |
| 220 | Biosynthesis of ansamycins | ko01051 | 11 | 0.000681959 |
| 221 | Caprolactam degradation | ko00930 | 11 | 0.000681959 |
| 222 | Flavone and flavonol biosynthesis | ko00944 | 11 | 0.000681959 |
| 223 | Glycosphingolipid biosynthesis - globo series | ko00603 | 11 | 0.000681959 |
| 224 | Pancreatic secretion | ko04972 | 11 | 0.000681959 |
| 225 | Benzoate degradation | ko00362 | 10 | 0.000619963 |
| 226 | Biotin metabolism | ko00780 | 10 | 0.000619963 |
| 227 | Bladder cancer | ko05219 | 10 | 0.000619963 |
| 228 | Dorso-ventral axis formation | ko04320 | 10 | 0.000619963 |
| 229 | Fat digestion and absorption | ko04975 | 10 | 0.000619963 |
| 230 | MAPK signaling pathway - fly | ko04013 | 10 | 0.000619963 |
| 231 | Other glycan degradation | ko00511 | 10 | 0.000619963 |
| 232 | Small cell lung cancer | ko05222 | 10 | 0.000619963 |
| 233 | Thiamine metabolism | ko00730 | 10 | 0.000619963 |
| 234 | Type I diabetes mellitus | ko04940 | 10 | 0.000619963 |
| 235 | African trypanosomiasis | ko05143 | 9 | 0.000557967 |
| 236 | Proximal tubule bicarbonate reclamation | ko04964 | 9 | 0.000557967 |
| 237 | Tetracycline biosynthesis | ko00253 | 9 | 0.000557967 |
| 238 | Amoebiasis | ko05146 | 8 | 0.00049597 |
| 239 | Bacterial toxins | ko02042 | 8 | 0.00049597 |
| 240 | Glucosinolate biosynthesis | ko00966 | 8 | 0.00049597 |
| 241 | Phosphonate and phosphinate metabolism | ko00440 | 8 | 0.00049597 |
| 242 | Carbohydrate digestion and absorption | ko04973 | 7 | 0.000433974 |
| 243 | Hedgehog signaling pathway | ko04340 | 7 | 0.000433974 |
| 244 | Jak-STAT signaling pathway | ko04630 | 7 | 0.000433974 |
| 245 | Lipopolysaccharide biosynthesis | ko00540 | 7 | 0.000433974 |
| 246 | Lipopolysaccharide biosynthesis proteins | ko01005 | 7 | 0.000433974 |
| 247 | Vitamin B6 metabolism | ko00750 | 7 | 0.000433974 |
| 248 | Basal cell carcinoma | ko05217 | 6 | 0.000371978 |
| 249 | Brassinosteroid biosynthesis | ko00905 | 6 | 0.000371978 |
| 250 | Butirosin and neomycin biosynthesis | ko00524 | 6 | 0.000371978 |
| 251 | Caffeine metabolism | ko00232 | 6 | 0.000371978 |
| 252 | Geraniol degradation | ko00281 | 6 | 0.000371978 |
| 253 | Leukocyte transendothelial migration | ko04670 | 6 | 0.000371978 |
| 254 | Neuroactive ligand-receptor interaction | ko04080 | 6 | 0.000371978 |
| 255 | Novobiocin biosynthesis | ko00401 | 6 | 0.000371978 |
| 256 | Phototransduction | ko04744 | 6 | 0.000371978 |
| 257 | Phototransduction - fly | ko04745 | 6 | 0.000371978 |
| 258 | RIG-I-like receptor signaling pathway | ko04622 | 6 | 0.000371978 |
| 259 | Salivary secretion | ko04970 | 6 | 0.000371978 |
| 260 | Synthesis and degradation of ketone bodies | ko00072 | 6 | 0.000371978 |
| 261 | Vasopressin-regulated water reabsorption | ko04962 | 6 | 0.000371978 |
| 262 | C5-Branched dibasic acid metabolism | ko00660 | 5 | 0.000309981 |
| 263 | D-Glutamine and D-glutamate metabolism | ko00471 | 5 | 0.000309981 |
| 264 | Gastric acid secretion | ko04971 | 5 | 0.000309981 |
| 265 | Hypertrophic cardiomyopathy (HCM) | ko05410 | 5 | 0.000309981 |
| 266 | Styrene degradation | ko00643 | 5 | 0.000309981 |
| 267 | Bisphenol degradation | ko00363 | 4 | 0.000247985 |
| 268 | Glycosaminoglycan biosynthesis - heparan sulfate | ko00534 | 4 | 0.000247985 |
| 269 | Glycosphingolipid biosynthesis - ganglio series | ko00604 | 4 | 0.000247985 |
| 270 | Lipoic acid metabolism | ko00785 | 4 | 0.000247985 |
| 271 | Olfactory transduction | ko04740 | 4 | 0.000247985 |
| 272 | Other types of O-glycan biosynthesis | ko00514 | 4 | 0.000247985 |
| 273 | Primary immunodeficiency | ko05340 | 4 | 0.000247985 |
| 274 | Fatty acid elongation in mitochondria | ko00062 | 3 | 0.000185989 |
| 275 | Indole alkaloid biosynthesis | ko00901 | 3 | 0.000185989 |
| 276 | Peptidoglycan biosynthesis | ko00550 | 3 | 0.000185989 |
| 277 | Bacterial motility proteins | ko02035 | 2 | 0.000123993 |
| 278 | Betalain biosynthesis | ko00965 | 2 | 0.000123993 |
| 279 | Biosynthesis of siderophore group nonribosomal peptides | ko01053 | 2 | 0.000123993 |
| 280 | Chlorocyclohexane and chlorobenzene degradation | ko00361 | 2 | 0.000123993 |
| 281 | Dilated cardiomyopathy (DCM) | ko05414 | 2 | 0.000123993 |
| 282 | Fluorobenzoate degradation | ko00364 | 2 | 0.000123993 |
| 283 | Malaria | ko05144 | 2 | 0.000123993 |
| 284 | Polycyclic aromatic hydrocarbon degradation | ko00624 | 2 | 0.000123993 |
| 285 | Polyketide sugar unit biosynthesis | ko00523 | 2 | 0.000123993 |
| 286 | Primary bile acid biosynthesis | ko00120 | 2 | 0.000123993 |
| 287 | Taste transduction | ko04742 | 2 | 0.000123993 |
| 288 | Toluene degradation | ko00623 | 2 | 0.000123993 |
| 289 | Vitamin digestion and absorption | ko04977 | 2 | 0.000123993 |
| 290 | Arrhythmogenic right ventricular cardiomyopathy (ARVC) | ko05412 | 1 | 6.19963E-05 |
| 291 | Bacterial chemotaxis | ko02030 | 1 | 6.19963E-05 |
| 292 | Circadian rhythm - fly | ko04711 | 1 | 6.19963E-05 |
| 293 | Ethylbenzene degradation | ko00642 | 1 | 6.19963E-05 |
| 294 | Glycosaminoglycan biosynthesis - chondroitin sulfate | ko00532 | 1 | 6.19963E-05 |
| 295 | Maturity onset diabetes of the young | ko04950 | 1 | 6.19963E-05 |
| 296 | Proteoglycans | ko00535 | 1 | 6.19963E-05 |
| 297 | Steroid hormone biosynthesis | ko00140 | 1 | 6.19963E-05 |
