# Supplementary material for: Transcriptome Sequencing and Expression Analysis of Terpenoid Biosynthesis Genes in Litsea cubeba
Source: PLoS One. 2013 Oct 9;8(10):e76890. doi: 10.1371/journal.pone.0076890 (PMC3793921; doi:10.1371/journal.pone.0076890)
Supplement: File S3 — The gene names, sequences and the primers used for RT-qPCR analysis. (DOC) [file pone.0076890.s003.doc]

1. Unigene 15981

acetoacetyl-CoA thiolase (AACT)

5’-GCTTTAAAAAACTTCAGTTTCCACTTCTGCTGACGCTTCGTTCGTGTCTTTTGCTATTCAATCTCTGCTTCTCTCGCTACCTCTCTCTCTTCTCGATCTCTCTACGAAGGTTCTTTTCTGAGTATTATCTGTTTTCTGATCATCTCATGGCTCCAGCTGCTGCTACAGATAATATAAAACCTCGAGATGTATGCATTGTTGGTGTTGCACGCACACCGATGGGAG-3’

AACT-F 5’-ACTTCTGCTGACGCTTCGTT-3’

AACT-R 5’-AGCTGGAGCCATGAGATGAT-3’

2. Unigene 4005

HMG-CoA synthase (HMGS)

5’-TGCTCGGCTGTACTTCAATGACTTTCTGAGGAATGCCAGCTCTGTTGGGAAGGATGCTATGGAAAAATTAGAGCCATTTTCATCTTTGTCTGGCGATGAAAGTTATCAGAATCGTGATCTTGAAAAGGTATCTCAGCTGGTTGCAAAAAGCCTTTATGATACAAAAGTACAACCATCAACTTTGTTACCAAAACAAGTTGGCAACATGTACACT-3’

HMGS-F 5’-TTCTGAGGAATGCCAGCTCT-3’

HMGS-R 5’-TGTTGCCAACTTGTTTTGGT-3’

3. Unigene 43919

HMG-CoA reductase (HMGR)

5’-CACCGGCCAAGATCCTGCACAGAACGTGGAAAGTTCCCACTGCATCACCATGATGGAACCTGTTAATGGTGGAAAGGATCTCCATGTCTCTGTAACAATGCCTTCCATTGAGGTGGGGACAGTTGGCGGTGGGACCCAACTTGCTTCTCAGTCAGCCTGCCTGAATCTACTTGGTGTGAAGGGTGCAAGTCTAGAATCACCTGGAGCAAATTCCAGGCTCTTGGCTACCATAGTTGCAGGATCTGTTCTAGCAGGAGAGCTCTCCCTCATGTCCGCTCTTGCTGCAGGGCAGCTTGTCAAGAGCCACATGAAATACAACAGATCAAGCAAAGATATATGCAAAGTTGTCTCCTGAGGAAAACCCATCTGGGAAAGAGACTTAAGAAAGTTAAATTTTAAAATAAAAGAAAAGGAATTCTTAGAGAATGAGGGATTAGATTCCAAGGATAATTCAGCAAAACATTCTGCAGATTCAGAAGGTAAAGGCCCAGTAGGATCCCCCTATTTTTCTATAACCATGTGGCCTTTCTCATCTGATAGCATTGTGAATGCCGAAAAAGAAGCCAGAAGGAAAAGGAGGGAAAGCTAAAGTGTCTTTTTGGGTTGCCATTTGTGAGGTGTGTGTAGGAGGCTCCCATTGCTTCTGGGCCTTCTTTGTTTCACTCTTGTTTTCATCTTATTAACCTTTTTCCATCTCTTGTGTTTGTTTTCTTGTATCTTGGAAGAAGACTATTCAATGGGGTTCCCAGCAGTTTAATTTTCTCATGTCTTTTCTGTCGTTGCAATGTATAGGACCACTGCAATAGTGAGATTTGTTGTACATTTTGTTTTCGACATTGGTCTTGGCTATGAATATCTCTACTATTCACTAATAATATATACTGCCCCCT-3’

HMGR-F 5’-AAAGTTCCCACTGCATCACC-3’

HMGR-R 5’-CAAGAGCCTGGAATTTGCTC-3’

4 Unigene 46543

mevalonate kinase (MVK)

5’-AAAGAAACCGTCAACACGAGCTCTTGTTTGTTTCTTCCATGGCATTCTTCTCTCCTTGGACAATGCACTTCTCATAAACCCTAGAAATCTAGGGTTTAAGAAGAAGAAGAGAGATGGAGATTCGAGCACGGGCGCCCGGAAAGATCATACTTTCCGGCGAGCACGCCGTCGTTCATGGAGCCACGGCGGTGGCCGCATCCATAGATCTCTACACGCATGTTTTGATTCGATCATCAACTTCCTCAGGAACCGATGATGGGCTTTTAGAATTGCAGCTCAAAGATATTGGGTTAGTGTTTTCGTGGCCAGTTCAAAGAATCAAAGAAACACTTCCAGAATTGGGTACTTCTCTTTCTCCATCATCAACGTCTTTCTTGCTAGAGTCCATGAAATCCATTGCTACTCTTGTTGAGGAGCAGAATATTCCTGAGGCTAAAATTGGACTCTTTGGCGGAGTTTCTGCCTTCCTCTTGCTGTACACCTCAATCCAAGGGTTTAAGCCTGCAACAGTAGTTATCAGCTCTGATCTTCCTATGGGTTCGGGTTTGGGTTCATCTGCTGCATTATGTGTTGCGCTTTCAGCAGCTTTACTTGCATATTCAGGTGCAGTTAGATGGGACGCCAACAACTATGGTTGGTTTACTCTAGCGGAGCTTGAGCTTGAATTGGTTAACAAATGGGCCTTTGTAGGTGAAAAAATCATTCATGGCAAGCCTTCTGGAATTGATAATACTGTGAGCACTTTTGGCAACATGATCATGTTCAGGTTGGGTGAATTAACTCGCATTAAATCTAATATGCCACTGAGAATGCTAATCACCAACACTAAGGTTGGAAGGAACACGAAGGCATTAGTTGCTGGTGTTTCTGAGCGAGCATCCAGGCATCCTGATGCTATGGCTGCTGTGTTTTCTGCTGTGGATTCCATCAGCAAGGAACTTTCTACCATTATCCAGTCGCCTGCTTCAGATGAACTCTCCATTACAGAGAATGAAGATAAGTTAGAGGAGCTTATGGAAATGAATCAAGGTTTGCTCCAGTCCATGGGAGTTAGCCACTCTGCTATAGAGGCTGTGATTCGAACAACTCTGAAGTACAAGTTAGCTTCAAAGCTGACAGGAGCTGGTGGTGGAGGCTGTGTTTTGACATTGTTGCCAGCATTGCTTGCTGATGCGGTTGTCGATAAAGTGATAGCTGAGCTTGAGTCATGTGGATTTCAGTGCTTGATAACTGCAATTGGAGGTAAAGGTCTTGAAATTTCTTCCTCTTGCAACTCCTCAACTTGTCATCCATCTGGAGAAATTTAAATGCTGATATCGAGGTAAGCTTTAGTACTAATTGGCAAAACTGGCAATGTGATATGGGCTTTTATTGCTATCTATTTTGTATTACTTGTGTACACTTTCTGGTTGGTTGCTTTCTGAGATTGTTATTATGTTTTGTTCCAGTTTAATTACCTTCCAATGTTGTTCACTAGTAGTTTCAGGCATGTGGGGAGCTCAGCAGTTTGTTTGAACCAAGCATATTATTCTGAATATCATTTCTTTGTTTTTT-3’

MVK-F 5’-TTATGTGTTGCGCTTTCAGC-3’

MVK-R 5’-GAAGGCTTGCCATGAATGAT-3’

5. Unigene 37221

phosphomevalonate kinase (PMVK)

5’-CAAAAAGAGTGAGAGACTCGGGTGCTGTTTGAAAAGCCTCTCTCGATCTGTTCTGCATCCCTGTCCAGTCTCGCCATCAACAGAAAGACGGAAAGCCTCAGGCCTCCAGGTTTCTCAGACTCAGCTACTCAGTTTGTGACTCACACAACTCAGACCAGACTCTTCTGTTAAGGTTGCAGGAAGTGAGGGCTGCAATGGCACAAGTAGTTGCGTCTGCTCCCGGGAAGGTTTTGATGACAGGTGGGTACTTGATTTTGGAGAGACCAAATGCTGGAATTGTTCTCAGCACGACTGCCCGTTTCTATGCAATCGTGAAGCCGTTTTATGAAGAAGTTACGCAAGATAGCTGGGCCTGGGCATGGACAGATGTGAAAGTAACATCTCCTCAGCTTTCTAGAGAGACCATGTACAAGTTGTCTCATAAGAACTCAACCCTCCACTGTATCTCTTCAAGGGACTCTGCCAACCCTTTTGTTGAACAAGCAGTACAATATGCAGTTGCAATTGCCACCACAATCTTAAATGATAGGGGAAAGAAGGATGAGTTACAGAAACTACTCTTGCAAGGCCTTGATATCACAATATTGGGTTGCAATGACTTTTACTCATATCGAAATCAGATTGAAGCACGTGGACTTCCTTTGAGGCCAGAAGCATTGGCCTCACTTCCACCCTTCTCATCAATTACCTTTAATGCAGAGGGGTCTGATGGGACTGTTACTAGAGATAATTGCAAACCTGAAGTTGCAAAAACTGGACTAGGTTCTTCAGC-3’

PMVK-F 5’-CTTCAAGGGACTCTGCCAAC-3’

PMVK-R 5’-GGAAGTCCACGTGCTTCAAT-3’

6. Unigene 57427

1-deoxy-D-xylulose-5-phosphate reductoisomerase (DXR)

5’-AAGATTCCCACTTTGCGATTCTCGTCCTATCCTTTCTCTGCCACTCTCTCTTGCCTTTTATAAGCTCTCACACATCTTCTTCTCTTTTCGTGTCTCTCTCTGTCTCTTTTTCTCTCATTTCCAGTTTCCTTCCTTTTTGGCTTTCCCCTTTTCGAGAAGAGATGGCTTTGAAATCCCCCCTGCACGTAGACATCGGAGGAATCTCCTTCTTGGATTCCAGCAAAGGAAGCCTTTGGAAGCTCAAAGGAGGATTTGTTTTGAAGAGGAAGGAGAATGGAATGCCACATGTAAGGTTAACCCGTTGTTCTGCCCAGGTGCCTCCACCGGCATGGCCAGGACGAGCCGTTGTAGAGCCAGGACGGAAGGTGTGGGATGGTCCCAAGCCTATCTCGATTGTTGGATCCACTGGTTCCATTGGAACTCAGACTTTGGACATAGTAGCTGAGAACCCGGACAAATTCAGAGTTGTTGCACTGGCAGCCGGTTCAAATGTGACCCTTCTTGCAGATCAGGTGAAGAGGTTCAAACCTCAACTGGTTGCTGTTAGAAACGAGTCATTAGTTGATGAATTTAAAGAGGCTTTGGCGGATGCTGAATACAAGCCTGAGATAATTCCTGGAGAGCAGGGTGTCATTGAAGTTGCACGTCACCCAGATGCTGTCACAGTAGTCACAGGAATAGTAGGATGTGCAGGTTTGAAGCCTACAGTAGCTGCAATTGAGGCCGGAAAAGACATTGCATTGGCAAACAAAGAGACTCTGATTGCAGGCGGTCCCTTTGTACTTCCTCTTGCACAAAAGCATAAAGTAAAAATACTTCCTGCTGACTCAGAACATTCTGCTATATTCCAGTGTATTCAAGGTCTGCCAGAGGGTGCACTACGGCGCATTATTTTGACTGCTTCCGGAGGGGCTTTCAGGGATTTGCCTGTTGAAAAACTCAAGGAGGTGAAAGTTGCTGATGCTTTAAAGCATCCTAACTGGACTATGGGAAAGAAGATCACAGTGGATTCTGCCACCCTCTTCAACAAGGGTCTTGAAGTTATTGAAGCCCACTATCTGTTTGGAGCTGAATATGATGATATTGAGATTGTGATTCATCCCCAGTCAATCATTCACTCAATGGTTGAGACCCAGGATTCATCAGTTCTTGCTCAGTTGGGATGGGCTGATATGCGCCTGCCAATTCTTTACACAATGTCATGGCCAGAGAGAATTTATTGCTCTGAAATCACCTGGCCTCGGCTTGACCTTTGCAAGTTGGGCTCTCTGACATTTAAGGCTCCTGATAATGTGAAATACCCATCCATGGATCTTGCTTACTCCGCTGGGCGTGCTGGAGGTACAATGACTGGAGTTCTTAGTGCAGCTAATGAGAAGGCTGTGGAGATGTTCATTAATGAGAAAATCGGCTACCTGGACATTTTCAAGATCGTGGAACTCACATGCGCCGAACACAGGAAAGAGCTAGTGACCAGTCCGTCCTTGGAGGAGATCATTCATTACGATTTGTGGGCTCGGGAGTTTGCTGCAAACTTAAATCTGTCTTCTGGGAAAAGACCCGTCCTGGCTTAATTGGTTGCAGCTGAAGAGGGCATGTCTAGTCTTGTGGGCCCCCATTGGACCCATCTATCCAATGGACAGATTTGCCAGGTATACATTCAGCTGTAAGCAAATGTAAAAAAGGCATCAGTGTTCCTTCAGATAGCTCTATAACAGAAAATAAATGAATGAATGCTAGTCCCTCTGTTGGAGGGATCCTTTTTCTGAGAAAAAAAAAAAAAAAAAAAA-3’

DXR-F 5’-TTTGGAAGCTCAAAGGAGGA-3’

DXR-R 5’-GCCAGTGCAACAACTCTGAA-3’

7. Unigene 55713

1-deoxy-Dxylulose-5-phosphate synthase (DXS)

5’-TGATGTGGACCTTCAGAAGCTTCCAGTTCGATTTGCCATGGACAGGGCTGGTCTAGTGGGTGCAGATGGCCCAACCCATTGTGGGGCGTTCGACACCACATACATGGCCTGTTTGCCAAATATGGTGGTGATGGCCCCATCGGATGAGACCGAACTGGTGCACATGGTTGCCACAGCAGCAGCCATTGATGACAGGCCCAGTTGTTTCAGGTTCCCCAGGGGAAATGGCATTGGATCCCCTCTTCCAACAAATTGCAAAGGAGAGCCCTTGGAGATTGGAAAAGGAAGGGTGGTGAGGGATGGAAATAAGGTGGCCATTTTAGGCTTTGGAACTATTGTACAGAACTGTGTAGCAGCAGGGAATGTACTCCAACAAGAGTTTGGAATTTCCATCACCATTGCTGATGCCCGTTTCTGCAAGCCTTTAGATCATCAACTCATCAGACAGCTAGCCCATGAGCATGAGATTCTCATCACTGTAGAAGAAGGATCAGTGGGAGGATTCAGTTCTCATGTGTCCCATTTCTTGGCCTTGAATGGACTCTTAGATGGAAAGCTTAAGTGGAGGTCTATGATGCTTCCTGATCGATATATAGACCATGGATCTCCTAAGGACCAGATTGAAGAGGCGGGGCTCACTTCTAAGCACATAGCAGCCACTGTTTTGTCTCTGATGGGCAAAACCAAGGACGGTCTCAAACTTCTTCATCGAAATCCAACTGCAGTGATGCTGTAACAGTATTTATTGGTAACGACTCTGGTGACAAGGAGAACTTGAAATTAGGAATGTTAATATAATGGGCTGTTTAGTACTGGCCCCAGATAGCCAAAAATAAAGACCATTTATTTAAGGTTGGGCATAAAATTGGGCTGAGCATGTGGACTAAAGGGTTGTGAATTACTGAGGTATATGTGTCACTTTTTTTTTTTTGTATGTAGTCTTGAGTCGAACATCAAGTAGGAGATCGTTATCAGGGTGATCTCTATATATATAGAAGGTATCTGTCTCCAGTTGTGTAAATATGTGTTCTCTTTGCAGTTTTAAGTGTACTATATATATATTTGAAGTTTGTCCACCCCATTATAGTTGTTACAAGGCTTTGCTGCTGCTTATAATGTATTTGA-3’

DXS-F 5’-TTTCTTGGCCTTGAATGGAC-3’

DXS-R 5’-CTGCAGTTGGATTTCGATGA-3’

8．Unigene 62474

4-diphosphocytidyl-2C-methyl-D-erythritol kinase （CMK）

5’-GTGTGTTCCTTTCAACATGGCTGCCACCCAATTCCTCTGCCAAAGCCTCTACCTTTCTTTCAAGAATGGGAAAAAACCCAAAAATCTTTGCAAGAGGAGCAGTTTTTCTCCTTCTTCTGGACATGGGTCATGCTTGTTCAATCAAAAGTCCCAAAATCAAAGATACCCAGTTGTCAGAGCTAGTGCTTCTGAATCAGCTGGAGGGAGAAAACAGGTGGAGCTTGTATATGATCTAGATGAAAAGATTAACAGATTAGCAGATGAAGTTGATATCAATGCTGGCCTTCAAAGGCTCTCTCTGTTCTCACCTTGTAAGATTAATGTTTTCTTGAGAATAACCAGAAAGAGGGAAGATGGGTTTCATGATTTGGCTTCTTTGTTTCATGTTATAAGTTTAGGGGATACAATTAAGTTTTCTTTGTCGCCAACAAAAACCAGAGATCGTATGTCAACAAATGTGCCTGGAGTCCCACTTGATGAGCGAAACTTGATAATTAAAGCACTTAATCTTTACAGAGAAAAGACTGGAAGCGACAACTTCTTTTGGATTCACCTAGACAAGAAAGTTCCTACTGGTGCTGGTCTTGGCGGTGGAAGTAGTAATGCTGCAACTGCATTATGGGCAGCAAATCAATTCAATGGTGGTATTGCAACTGAACAGGAGCTTCAAGAATGGTCAGCTGAAATTGGTTCGGACATTCCTTTCTTTTTCTCACATGGAGCAGCATATTGTACCAGTAGAGGTGAGGTTGTTCAAGATATTCCACCACCAATACCCTTGGACCTCCCAATGGTTCTTATAAAACCACCTGAGGCATGCCCGACTGCTGAAGTTTACAAGCGGTTTCGGCTGGATCAAACTAGCTCAGTCAACCCTTTGACCTTGCTGGAGAAGATCAAACAAGCAGGGATTTCTCAAGATGTTTGTATCAATGATTTGGAGCCCCCTGCATTTGAAGTCTTACCGTCTCTGAAACGGTTGAAACAACGGATTCATGCCGCAGGTCGCGGACAATATGATGCGGTTTTTATGTCAGGAAGTGGAAGCACTATTGTTGGACTCGGTTCTCCAGATCCTCCCCAGTTTATCTATGATGAAGATGACTACATGGATGTATTTGTATCTGAGGCTCACTTTCTCACTCGGGGAGAGAACCAGTGGTACACAGAACCAGGCTCAACATTCAGTTCTTCTGACTCACCCGTCACCGACCGCTTGTCGGTTTAAGTAATGTCTATTGTTGTTTTGATGGAATACAATAAAAAAAAAATATTGGTTGGGTACCTTGTTTTCTTTTTATGGATCCAAAGCAGACTTTTTCTCTGTGTGCTATTCCCCATCTTTCAAGTGGGGTTATAGATAAGGGCAGGCATTTGTTCATGAGATAGAAATTGGGTTATGAATTTCTTACAGATGAATTTAGTATCTTGCATAAAAGACCAAAGAGATTACTCTAAAAAAATGGTGCCATCTTTCAAGTTGGGGCAT-3’

CMK-F 5’-GGCCTTCAAAGGCTCTCTCT-3’

CMK-R 5’-GGACTCCAGGCACATTTGTT-3’

9. Unigene 60959

1-Hydroxy-2-methyl-2-(E)-butenyl-4-diphosphate Synthase (HDS)

5’-ATTTTTTACTCGCCAAACGTTTGTCGTCCCTTCCCTCCATAAAATCACATTTCCTTGAAAAGCAGCTCAAAAAACACACGCACACAAATCCAAACCATCCGAATCCCCTTCCACCACTAAATTCCATCTCCAGATCCTTCGAGTCGAAAGCGATTCCTCTCCTCCTTCTTCATCCGCTTCTTCTTCTGCAGAAGAAGATTGTTCTAGAACTGTGAAGGTCAGGTAAGGGATTCGTAGAGATGGCTACTGGGTCAGTCCCGGCTTCTATTTCTGGTTTGAAGACTAGGGATCATGGTTTAAGCTTCGCAAAAAGTGTAGATTTTGTGAAGGTTACATGCTTGCCTTCACAGAAGATCAAGCCTCAGAGAGATAATATATCTGTTATCAGAAACTCAAAACAAGGTCCTGAAACCATTGAGTTGCAGCCTGCTTCTGAGGGAAGCCCTTTGCTAGTTCCTAGGCAGAAGTACTGTGAATCAGTGCACAAGACTATCAGGAGAAAGACTCGGACTGTGATGGTAGGAAATGTAGCTCTTGGTAGTGAGCATCCTATAAGGATTCAGACGATGACTACTAGCGATACAAAAGATGTTGCCGGAACTGTTCAGGAGGTAATGAGAATAGCGGACAAGGGAGCAGATATTGTTCGGATCACAGTTCAGGGGAGGAAAGAAGCAGATGCGTGTTTTGAAATAAAGAACACCCTAGTCCAGAAGAATTATAACATTCCTCTGGTGGCCGACATTCATTTTGCTCCTCCTATCGCTTTGAGAGTTGCTGAATGTTTTGACAAGATTCGGGTCAACCCGGGAAATTTTGCTGATAGGCGGGCTCAGTTTGAGACGCTGGAGTATACTGAAGATGATTATCAAAAGGAACTTGAGCATATTGAGCAGGTTTTCTCTCCATTAGTTGAGAAATGTAAAAAGTACGGAAGAGCAATGCGCATTGGAACAAATCATGGAAGTCTTTCTGACAGGATTATGAGCTACTATGGGGATTCTCCTAGGGGGATGGTTGAATCTGCCTTTGAGTTTGCAAGGATTTGCCGCAAGTTGGACTTCCACAACTTTGTCTTCTCAATGAAAGCAAGCAACCCAGTAATCATGGTTCAAGCATACCGCTTACTGGTTGCAGAAATGTTTGTTCAGGGTTGGGACTATCCTCTACACTTGGGAGTCACTGAAGCTGGTGAAGGTGAGGATGGACGGATGAAATCTGCAATTGGCATTGGAACCCTTCTTCAGGATGGTTTGGGGGATACAATCCGTGTTTCCCTCACAGAACCACCAGAGGAAGAGATAGATCCTTGCAGAAGACTAGCCAACCTTGGCATGCAAGCTTCAAAGCTTCAGAAGGGGGTGGTACCATTTGAAGAAAAACACAGACGTTATTTTGATTTTCAGCGTAGAACTGGTCAATTACCAATTCAGAAAGAGGGTGAAGAGGTAGACTACCGAGGAGTCTTGCACCGCGATGGTTCTGTTCTTATGTCTGTGTCTCTAGATCAGTTGAAGACACCCGAACTCCTCTATAGATCACTTGCAGCGAAACTTGTTGTTGGCATGCCATTTAAGGATCTGGCAACAGTTGATTCAATTCTTGTGAGAGAGCTTCCTCCAGTAGAAAACACTGATGCTAGGCTTGCACTCAAAAGGCTGATAGATATAAGCATGGGAGTTTTGACTCCATTGTCAGAGCAGCTTACGAAGCCCTTACCTAATGCCATGGTCCTAGTGAATCTTAAGGAACTGGCAAGCGGTGCCCACAAACTTTTACCAGATGGCACGCGCTTGGCAGTTTCTGTTCGTGGTGATGAACCCTATGAGGAGCTGGATATACTTAAAGACGTGGAAATAACAATGCTTCTACACAATCTACCATTTTCTGAAGAGAAAAACAGCAGAGTACATTCAGCAAGGAGGCTGTTTGAGTATCTGTCAGACAATTCTCTGAACTTCCCTGTAATTCACCACATGCAGTTTCCTGAAGGGATACACAGAGATGATCTAGTGATTGGTGCTGGAAGCAATGTGGGAGCCCTTCTGGTCGATGGCCTTGGAGATGGCTTATTCTTGGAGGCTCCTGATCAGGACTTTGATTTTCTGAGGAACACATCCTTCAATTTGCTCCAAGGTTGCAGAATGCGAAATACAAAAACGGAGTATGTTTCATGCCCATCCTGTGGTCGTACACTGTTTGACCTCCAAGAAGTGAGTGCTGAGATAAGAGAGAAGACAGCCCATTTGCCTGGTGTTTCGATTGCAATCATGGGTTGCATTGTTAATGGGCCGGGAGAGATGGCAGATGCAGATTTTGGGTATGTTGGAGGTGCTCCAGGAAAGATTGACCTCTACGTTGGGAAGACTGTGGTGAAGCGAGGGATTGAGATGGCGCATGCCACCAATGCATTGATTCAGCTGATTAAAGACCATGGCCGCTGGGTGGATCCACCAGCTGATGAGTAGATCCAGTATTCCCCATTTGGTGAATGCTAGTGGAAGAGAGGTGTATTTAGTGACGAAGCTCCATCCAGTATACATAGATATCTTACATTGTCCTCAACACCAGAAGCATATACAACTGTAAACAGCAGGAAATTAAGTGAGGGAAAATTCTCTTTGTGAGCTGCACTGTGCAAAGCTCTAATGCATAATCAGTTTTTCCCTAATACATGATTGGGAAATTGATACCCTGATAGATATGTATTTGTCCTTTTTTTATTAATTGAGAAAATAAGACTTTTCAGAGAAAACAAACATCTGAGCTCAGCTTGATAGTAACCATTGCAGGCAAGCAGATTTTCGCCAAATGTGTGGGTGTTTTATTGAAAAAAATTATCAGCTGTATTAGTGTAGGTTTTAAATATTGCGAAGCCCCT-3’

HDS-F 5’-AATCCGTGTTTCCCTCACAG-3’

HDS-R 5’-GGTGCAAGACTCCTCGGTAG-3’

10. Unigene 56508

1-Hydroxy-2-methyl-2-(E)-butenyl-4-diphosphate Reductase (HDR)

5’-CAACAGCACCAAACATCAGAAAACGACAAAACGCGCGCTCGTAGTAGAAAAAACGGATGGCTACGAGATAAAATGCGCATCTTCTCCAAAAACACACTCACACAACTTACATCTGCAACGGCATCTTCTTCGTCCCGCTGCTAACGTGCGCCAGCTTCCTCCGGTTTCCATCCATGGCGACGGCTCTGCAATTCACGCTCTCCTCCCACCGCTCCGACCTCCTCCTCGCAAAAGCTACTTTTGGGATCCGATCCGGGACCTGGAAACCGATCGCCACCGTCCGATGCTGCAGCAGCACCGAATCCGCCGTCGAGTCCGAGTTCGACGCCAAGGTGTTTCGGAAGAACCTGACACGAAGCAAGAACTACAATCGGAAGGGTTTCGGACACAAGGAGGAGACGATGGAGCTGATGAATCAGGAGTACACGAGCGATGTGATAAAGACGCTGAGGGAGAACGGGAACGAGTATACGTGGGGAAACGTAACGGTGAAGCTGGCGGAGTCGTACGGATTCTGCTGGGGTGTTGAACGTGCGGTCCAGATCGCGTATGAGGCAAGGAAGCAGTTCCCGGAGGAGAAGATCTGGATTACCAACGAGATTATCCATAATCCAACTGTTAATAAGAGATTAGAAGAGATGGACGTCAAGAATATTCCAATTGAGGATGGGAAGAAACAATTTGATGTTGTTGAGAAGGATGATGTTGTGATTTTACCTGCTTTTGGAGCTGCTGTGGAAGAGATGTTGACTTTGAGCGAAAAGAACGTACAAATAGTTGATACAACTTGCCCATGGGTGTCTAAGGTCTGGAACACTGTTGAGAAACACAAGAAGGGGGAGTACACTTCAATAATTCATGGTAAATATTCTCATGAAGAGACAGTTGCAACTGCATCTTTTGCAGGGAAGTATATTATTGTTAAGAACATGGCAGAGGCAATGTATGTATGTGATTACATTCTTGGAGGTAAACTTGACGGATCTAGTTCAACAAAAGAAGAGTTTATGGAGAAATTCAAATATGCAGTTTCCAGCGGGTTTGATCCAGACATTGATCTAGAAAAGGCTGGCATTGCAAATCAAACTACAATGCTCAAGGGAGAGACTGAAGAAATTGGTAAATTGGTTGAGAAGACAATGATGCGCAAGTATGGGATAGAAAATATCAATGAGCACTTCGCGAGTTTCAATACGATTTGTGATGCTACACAGGAGCGACAAGATGCAATGTACAAGTTGGTGAAGGAGAAACTAGATCTCGTGATAGTGGTTGGTGGATGGAACTCCAGTAACACCTCTCATCTACAAGAGATTGCAGAGCACTATGGAATTCCATCATACTGGATCGATAGCGAAAAGAGAGTAGGACCAGGAAACAGAATAAGCTACAAGTTGAATCATGGAGAGCTGGTTGAGAAAGAGAACTTTTTACCAAAAGGTCCCATCACAATTGGGGTAACTTCAGGTGCTTCGACTCCAGATAAGGTAGTTGAGGACGTCCTGAACAAGTTGTTCGACATCAAACGTGAAGAAGCTTTACAGTTAGCCTAAATCTATGCCTGATCGAAATTTCAATAAACCCGCAGTCCCTCCAATCTAAGAAAAAATTGTTCGATGACTTGGTGGATCCCAATGGGTGTTGGTTATCTGCTTCATATGTATAGCATCTATAGCCTGCAGTCCTTTACAGATTGGGGAACTTTTAAGTGGTTATCTGTATACAAAAATATCAATAGCGGTTGAAAGAAATGCTGGCTTGACTCATAAAATGTGAAATGAGTTACAATGAATGGATAAAATTTAGTTACCATTCCAAAACAGAAGAAAGAAAAAAGATGATGAATTAGAATTGTATACTTTCAGAATTATGCTTTTAATATATCAGTAGTGCGTTAAAGGAACACCAACTTTTAAAAGTGTGAATGGAATCTGTAGTTGCTGTGTGACAAGAAGCATTGCTGC-3’

HDR-F 5’-TGATGCGCAAGTATGGGATA-3’

HDR-R 5’-TCCACCAACCACTATCACGA-3’

11. Unigene 4493

farnesyl diphosphate synthase (FPPS)

5’-TGCCCCGTTTCTTTCTTCTTCTCTCTCGATCCATCTTTTCTCTTCTCTGCTAAAAGCGATCGGCTTCGTCTTCTTTCTTTGTAATCGATGGCTGCAGCGCCAAATGGAAAGGCAGCAGATCAGCGTTCGGAGTTTCTGAGGGTGTATGATCGGCTAAAGGCCGACCTCCTCCAAGACCCAGCTTTCGATTTCACCGAAGATTCCAAGAACTGGGTCGATCGAATGCTGGATTATAATGTACCAGGAGGGAAGCTGAACCGCGGGTTATCTGTGATTGATAGCTACCAATTGCTTAAAGGTGGAAAGGAACTGACACCAGAAGAATACTTTGATGCATCTGTTCTCGGTTGGTGCATCGAATGGCTTCAAGCTTATTTCTTGGTCCTTGATGATATTATGGATGAATCTCATACAAGGCGTGGCCAGCCTTGTTGGTTCAGAAGACCAAAGGTTGGTATGATTGCTGTGAATGACGGTATCGTACTTCGTAACCATATTCCTAGAATGCTCAATAAACAATTCAAGGGAAGGCCATACTATGCTGATCTCCTTGATTTGTTCAATGAGGTTGAGTTCCAGACAACTCAAGGACAAATGCTTGATCTGATCACCACTCTTGATAAAGAAAATAATCTGAACAAATACAGCTTGCCAGTTTATCGGCGCATTGTAACATACAAAACTGCCTTCTATTCATTTTATCTTCCAGTCGCATGTGCTTTGCTTCTGATGGGTGAGAGCTTGGACAAACACCTTGATGTGAAGAACATTCTCATTGAAATGGGAGTTTACTTTCAAGTTCAGGATGATTATTTGGATTGCTATGGTGATCCTAAAGTAATCGGCAAGATCGGAACTGATATTGAAGATTACAAGTGCTCTTGGTTGGTTGTGAAAGCTCTTGAAAGGGCCAACGAGCACCAAAAGAAGATACTATATGAGAACTATGGAAACACAGATTCAGAAAAGGTAGCCAAAGTGAAGGCTCTTTACAACGAACTTGGTCTCCAGAATGTATTTTTGGAGTATGAGCGAGAGAGTTATGCAAAGCTCATCTTGTCCATTGAAGCCCAACCTAGTAAAGCAATGCAAGAGGTGCTGAAGTCCTTCTTGGGAAAGATCTACAAGAGGCAAAAGTAGAGAGTTCAAAGGGAAGCAAACTCTACAGAGGAATGGAATAGATTGGAGATCACAACTATAAAAATTTTTCCCTGATATTTTTAGTTGGTATGGTTTGTTCATCTACATATTTATGATACGCCAGCATGTGTTTGACATGGCTGATTTCAGTGTTTTTCTTATAGTTTGAAATGTCCTCAACAATAATGTTCAGTAAGGGAAATAAAGAGTAGTAATTTAGCCGTTCCAAAAAAAAAAAAAAAAAAAA-3’

FPPS-F 5’-TCGATTTCACCGAAGATTCC-3’

FPPS-R 5’-GCCACGCCTTGTATGAGATT-3’

12. Unigene 57598

geranyl diphosphate synthase (GPPS)

5’-GCACCACCAGGAGTTTCTCTGGTGAAAAGCCTCTGAAATTCTGTTCTGGTTTCCCTGTTGCCTCAAAGGTTTTTGGTTGCAGAGGAAGTTATTGTTATGGTTCGCATGCCTACCATGATGTAAGGTATCAAATTCATCAAGAAAGAAGCTCTGCGGTTGAGGAACCTTTGGATCCATTTTCTCTTGTTGCTGATGAACTTTCAACCCTAGCTAATAGATTACGTTCCATGGTGGTTGCTGAGGTACCTAAGCTGGCATCAGCTGCTGAATATTTCTTCAAGATGGGTGTTGAAGGAAAGAGATTTCGTCCCACGGTGCTATTGTTGATGGCATCAGCTTTAAATATGTCCATACCCGAATCAGTTGCTGAAAGTGTGCTTCATGGCATGTTGAAGGAAACGCGTGCAAGGCAGCAGTGTATTGCTGAAATCACTGAAATGATTCATGTTGCAAGCCTTCTTCATGATGACGTCTTAGATGATGCAGATACAAGACGTGGTATCGGCTCTTTGAATTTTGTAATGGGCAACAAGATTTCTGTACTCGCGGGAGATTTTCTGCTTTCCAGAGCTTGTGTTGCACTGGCATCATTGAAAAATACCGAGGTTGTGTCATTACTGGCTACTGTTGTGGAGCATCTTGTTACTGGTGAAACAATGCAAATGACAACTACGTCTGATCAACGTTGTAGCATGGAGTACTACTTGCAGAAAACATACTATAAGACAGCATCATTGATATCTAACAGCTGCAAAGCAATTGCTCTTCTTGCGGGGCAAACAGCCGAAGTTTCAATGCTTGCTTATGATTATGGTCGAAATCTGGGATTGGCGCATCAATTGATTGATGATGTACTTGATTTCACTGGCACATCTGCTTCCCTTGGAAAGGGTTCCTTATCTGACATCCGCCATGGAATTATAACAGCTCCAGTATTATTTGCCATCGAAGAATTCCCTCAACTACGTGGATTGGTTGATCGAGGATTCGACAACCCTTCAGATATTGATCTTGCACTTGACTACCTTGGAAAGAGCCAAGGGATACAGAGGACAAAGGATCTTGCATCCAAACATGCTAGTCTTGCTGCTGCAGCAATTGATTCTTTCCCTGAAAGTGACGATGAGGATATTCGGATTTCTCGGCGGGCACTTGTTGATCTTACTCAGAGAGTCATTACAAGAACGAAGTGAGAGAGAGACACCCCCTCAACGAAGAAAATAAGGAATTCTTTTGCTAAAATATTCTTTTTATTATTCTTAAAGCATTGCTTATTTTTGTAGCCCCAGTCTCAAGCCTGAGGATCCGGAATTTCACATGTAAATGTATAAATTTCATGTTGTCCTTTTTTGTCTGAATTTATTTCCCTCTATGATCCAGCAGAAATTCTGCAATCATTCTTCATTTCCAGCCACCGATGAGGTCGAATTCTGTAGCTCATGTGAAACACCCAGCAGAGATTTGCTAATTTCTCATGTTGCGCCGATCCATCAACTAGGTGCATTCTGTGGATTGATGGGGTTGGTATCATCTGAGTGTAGAAGATTTTGAAACGTCTGAGTCGACCCACTGGTCTGATGGTCTTGTATCATCTTAATAGTTTCAAATGTTTCTCCAACTTAATCTTCTATTTTATGATTGTAATTAGTCTTGGTGAATGAATAATCAATTGAATTTTCTTTCCATAAGAACAACTAGAAGTGTTCAACTAGGCACAAGAAGGGCAACTTCTTGGAAAACGTATTCCTTTCCATGGATAAAAGTGGTAAAAGCAAGTACACAAGTGTGAACTATTGGTCCCAGTGGGACTAGTAGATTTGAATATTATGACAGCT-3’

GPPS-F 5’-GTTTCCCTGTTGCCTCAAAG-3’

GPPS-R 5’-GGTACCTCAGCAACCACCAT-3’

13. Unigene 50889

isopentenyl-diphosphate isomerase (IPPI)

5’-AGAGGAGAGGAGAGGAGAGGAGAGGAGAGGAGAGGAGAGGAGAGGAGAGGAGAAAAATCCACATTGTAATTTTCTTTGTTTCCTCAAAAAAAAAAAAGAAAGAAAATGGCAGCGTCTTCTCCTTCGGTCTGCTCTCTTCTCAACATCGCATCAAGAGGAACCGCCGGCGCCTCTACAGTCTCTTCTCCAGCATTAGCATCCACTTCCCGTGTGCGTCTCATACCATGCGACACCACCTTCAAAGGAGTAGGAGGCGGCTCACTGCTTCCATTTCCATGTTCCTCTCCCACGCGCGTCTTCTTCTCCGTCCGAACGCCGATTTGGAAATGCTCATCCTCCTCCTCCTCCTCCTCTTCAGTTGCCACCGGCAAAGAAGCTTTAGACTCTTCCATGGACGATGTCCAGAGGCGTCTCATGTTCGAAGACGAGTGCATCTTGGTTGATGAGAAAGACTATGTCATTGGGCACGACTCTAAATATAATTGTCATTTGATGGAAAAAATTGAGTCTGAGAACTTGCTACATCGAGCTTTTAGTATTTTCCTCTTTAACTCAAAATATGAGTTGCTCCTTCAGCAAAGGTCTGCAACCAAGGTAACATTTCCTGAAGTGTGGACAAACACCTGCTGCAGCCATCCACTTTATCGTGATTCTGAGCTCATCGAGGAGAACTTTCTCGGGGTAAAGAATGCCGCACAAAGGAAACTTTTTGATGAACTTGGCATTCCTGCTGAAGGTTTACCCACCGACAAGTTCATTCCCGTAGGTCGTATACTGTACAAGGCCCCCTCCGATGGCAAGTGGGGCGAGCATGAATTGGATTATTTACTCTTCATTGTAGTCGACGTCGAGTTGAATCCCAACCCTGATGAAGTTGCGGATGTCAAGTATGTTAATAGGGACCAGTTGAGAGAATTGTTGAGGAAAGCGGATGCTGGGGAAGATGGCATCAAGCTATCACCTTGGTTCAAATTGGTGGCTGATAATTTCCTTTTCAAGTGGTGGGACCATGTTGAAGAAGGCACTCTTCAGGAAGCCGCTGACATGAAAACCATACATAAGTTGTAAATATATGAATGTATGTATAAACTTTTAGCAAAAATAAAATGTTTGTATAAAAAACAT-3’

IPPI-F 5’-CTCATACCATGCGACACCAC-3’

IPPI-R 5’-TCGTCTTCGAACATGAGACG-3’

14. Unigene 46190

mevalonate diphosphate decarboxylase (MDV)

5’-CTTCTTCTTCTTCTTCTTCTTCCCGACACACTCCATTTTGAACTCCCATTCTCATTCTCCTGCTACTACTACTATCCTTCAAATCCCAATCGATCTTGAAACCCTATCATTCCAAACACTCTTGTCCTTTATCTTTCCATTGAAATCCCCTCCGAGATCGGAACCTTAGAGAATCTGAGATTTTAGGGTTTTTGGAGCTCTAGATCCCACTTTTTTTTTTTTCATTTTCATGGCGGTCTCGAATGGACCTGAGCCATGGATGTTGATGGTAACAGCTAGATCTCCGACGAACATTGCCGTGATCAAGTACTGGGGAAAGCGCGACGAAACCCTCATCCTCCCGATCAATGACAGCATCAGCGTGACGCTGGATCCGGACCATCTGTGCACGACCACCACCGTCGCGGTTAGCCCGGCGTTTGATTCTGATCGCATGTGGCTCAATGGAAAAGAGATTTCTCTCAGCGGAGGGCGGTACCAGAGCTGTTTGAGGGAGATCAGGAGGAGGGCATGCGATGTAGAAGACGAGAAGAAGGGGATTTGGATTAGGAAAGAGGATTGGGAGAGGCTACGGGTGCATATAGCTTCTTACAATAACTTCCCTACTGCGGCCGGATTGGCTTCCTCTGCTGCAGGATTTGCTTGCCTGGCATTTGCGCTTGCAAAACTGATGAATATTAAAGAAGAAAATGGAGAACTTTCATCTATTGCAAGGCAAGGTTCAGGCAGTGCTTGTCGCAGTCTATATGGTGGATTTGTAAAATGGGTTATGGGAAATGATGCTTCTGGACATGACAGCATTGCAGTTCAACTTGCAAATGAGTCACACTGGGATGATCTTGTTATTATTATTGCAGTAGTAAGTTCACGACAGAAAGAAACAAGTAGCACTGCAGGAATGCGCGAGAGTGTTGACACAAGTACTCTTTTACATTATAGATCCAAGGTGGTGGTTCCACAGCGCATATTGCAAATGGAAGAAGCCATTAGAAATCGGGATTTTCAGTCTTTTGCAAGGTTGACTTGTGCAGACAGCAATCAGTTTCATGCCGTTTGCTTGGATACCTCTCCTCCCATATTCTACATGAATGATACTTCCCGCAGGATAATAAATTGTGTGGAAAGATGGAATCAGTTCGAAGGAACACCACAGGTGGCTTACACTTATGATGCGGGGCCCAATGCAGTTCTTATTGCGCACAACAGAACAGTTGCTAGTCTTCTGCTACAGAGGCTGCTCTTCTACTTTCCTCCTCCCCCTGACACTGAACTAACTAGCTATTTATTAGGTGATAAATCAATATTGGAAGAAGCCGGTTTGCAGACAATGAAAGATGTGGAAAACTTGCAGGCACCTCCAGAAATAAAGGGCAGCATTTCTGTTGATAAAAATTCTGGCAGTGTCAGTTATTTCATCTGCACGAGACCTGGGAGAGGTCCAACATTGCTTGTTGATGAAGACCAAGCCCTCATTGATCCCAAAACTGGGATTCCTAAGTCGTGTTAAAACACCATTTTTCTTGTTCCATATTTTGGCTTCTTTCTTCTTGAATCTGCCACCTGCAATCTTGTAGTTGCAGCAACGTGCACCTTCTGATGGTGCAATGAGCTTTTGTTGAAGTAGGAAAACAGGACCATATTTTGGCTGTTTTGATGAGTTTGCAAGACAAAGCCATGGGTCTCTAGTTGATGGGATTGATTGTTTGGAGAATATGCTGGATTAAGGGGTGAAATGATCTTGTGAGCGGATCAAGTTGGTTATATGCCCTTTTACCGGTTCATTCCTTTTGTATTATTTTTTCTTGTTATTGATGTTGGAG-3’

MDV-F1 5’-GGAAAGAGGATTGGGAGAGG-3’

MDV-R1 5’-ACAAGCACTGCCTGAACCTT-3’

15. Unigene 38014

Geraniol synthase (GES)

5’-GCTTGTATGAAGCTTCACACCTTGCCTTTCAAGGGGAGACTATCTTGGATGAGGCAAGAGCTTTCACAAGCACACATCTCATGGATATCCGGGAGAACATAGACCCAGTCCTTCATAAAAAAGTAGAGCATGCTTTGGATATTCCTTTGCATTGGCGATTAGAAAAATTAGAGGCTAGGTGGTACATAGACATATATATGAGGGAAGAAGGCATGAATTCTTCTTTACTTGAATTGGCAAAGCTTCATTTCAACAATGTGCAAACAACATTCCAAAGAAGTTTAAGGAGTGTGTCAAGGTGGTGGAAAGATATGGGTCTTGGTGCGCAGTACTCTAGCTTTGCTAGAGACAGGTTGGTGGATAGTTTCTTTTGGGCCGCTGCAATGACACCCGAGCCACAGTTTGGACGTTGCCAAGAAGGTGTTACGAAAGTTATTCAACTCATATCAACAATTGATGATATCTATGACGTGTATGGTACAGTGGATGAGCTAGAACTTTTTACTAATGCGGTTAATAGATGGGATCTTAAGGCAATGGAGCAACTTCCTGAATATATGAAG-3’

GES-F 5’-ATGAGGGAAGAAGGCATGAA-3’

GES-R 5’-AGCGGCCCAAAAGAAACTAT-3’

16 Unigene 1894

Trans-ocimene synthase (OS)

5’-TTCTTCGACAACATGGTTATGGTGTTACTCCAGACATTTTCAACAAGTTTTTGGAGAAGGAAAGGACATTCAAAGCATGCACAAGCCTAGATGCAAAAGGCCTTTTGAGCCTATATGAAGCATCACATACTATGATACATGGTGAGGAAGTGTTGGAAGACGCCAAGGAATTCAGTGTTAAGCATCTTAACTACTTGATGGGGAACTTACAGAACAATCTAAGAGAACAAGTGCAACATGCCCTAGAAATGCCCTTGCATTGGAGGATGCCAAGGCTAGAAGCAAAGCATTATATAGATGTGAATGGGAGGTCAGATGAGAGGAATATGGTTTTACTAGAGCTGGCAAGGTTGGATTTCAATTTTGTGCAGTCCAAGCACCAAGAAGAGCTAAAGGAGGTGTCAAGATGGTGGAGAGACTTGGGTCTTGCAAAGAAGTTGGGATTTTCTAGGGT-3’

OS-F 5’-CAAGTGCAACATGCCCTAGA-3’

OS-R 5’-GCTCTTCTTGGTGCTTGGAC-3’
